# Supplementary material for: A fully solution-processed organic microcavity laser in the strong light-matter coupling regime
Source: Nat Commun. 2026 Jul 3;17:8280. doi: 10.1038/s41467-026-75118-1 (PMC13469614; doi:10.1038/s41467-026-75118-1)
Supplement: Supplementary file 1 [file 41467_2026_75118_MOESM1_ESM.pdf]

Supplementary Information

A fully solution-processed organic microcavity laser in the strong light–matter coupling regime

Hassan A. Qureshi, Henri Lyyra, Akseli Korkeamäki, Oskar Tuomi, Antti J. Moilanen, and Konstantinos S. Daskalakis
Corresponding author: konstantinos.daskalakis@utu.fi

CONTENTS

Supplementary Figure 1 Reflectivity spectrum of the dielectric distributed Bragg reflector (DBR).
Supplementary Figure 2 Power-dependent emission from the neat DPAVB:PVA film, showing the onset of amplified spontaneous emission (ASE).
Supplementary Figure 3 Power-dependent photoluminescence and linewidth for different combinations of excitation and collection polarizations.
Supplementary Figure 4 Time-resolved photoluminescence decay traces of the microcavity emission at various excitation powers.
Supplementary Figure 5 Power-dependent emission of microcavities at different cavity–exciton detunings.
Supplementary Figure 6 Real-space images of neat DPAVB:PVA films under increasing pump fluence, showing irreversible damage associated with ASE cycling.
Supplementary Figure 7 Real-space images of the polariton microcavity under repeated threshold cycling, demonstrating fully reversible radial emission and absence of damage.
Supplementary Figure 8 Photograph of the microcavity sample and spatially resolved PL spectra demonstrating detuning variation due to the spin-coating thickness gradient.
Supplementary Figure 9 Transfer-matrix simulations of full and empty cavities and measured optical constants of the DPAVB:PS film.
Supplementary Figure 10 Angle-resolved reflection and emission below and above threshold at different pump powers.
Supplementary Figure 11 Power-dependent blueshift and angle shift of the emission maximum and their correlation for three detuned cavities.
Supplementary Figure 12 Power-dependent real-space emission profiles, ring-radius analysis, and corresponding blueshift for three detuned cavities.
Supplementary Figure 13 Fit sensitivity analysis.
Supplementary Figure 14 Spatial dependence of fit temperature.

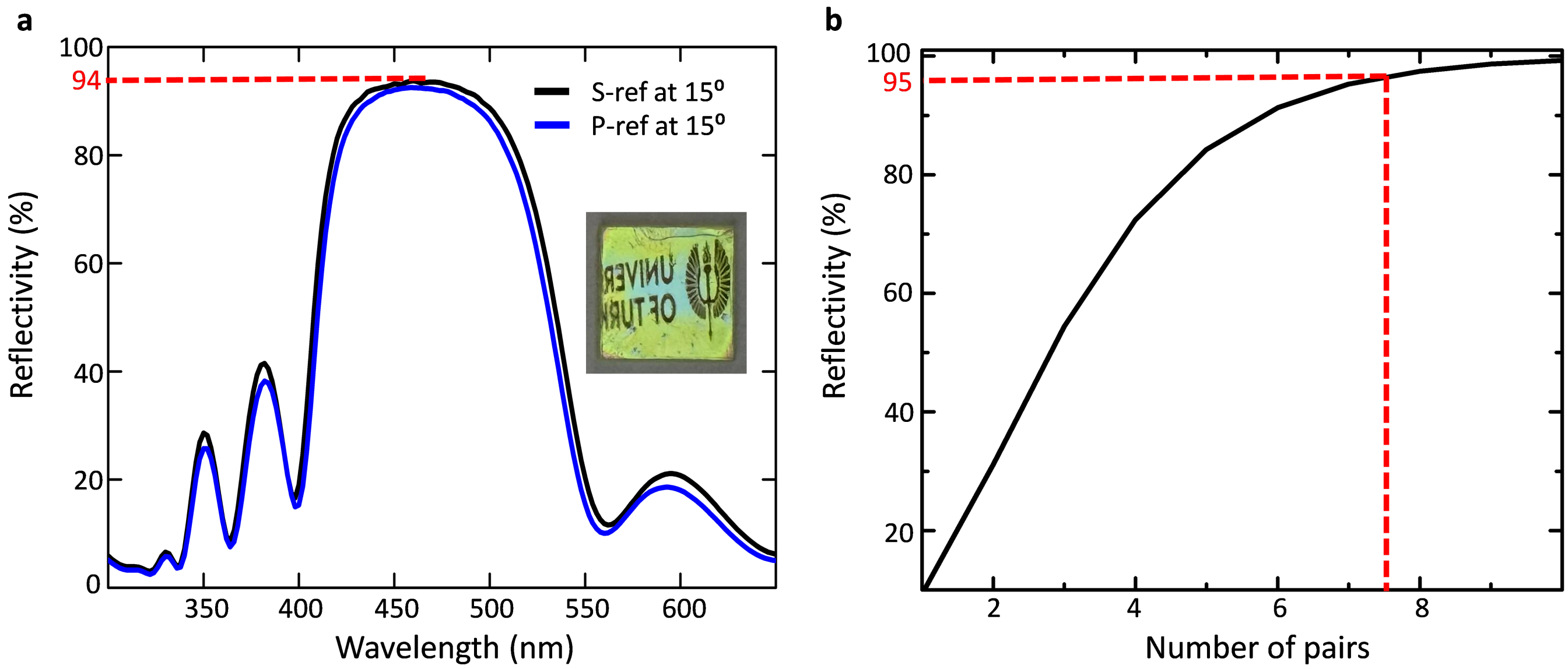

Supplementary Fig. 1. (a) Reflectivity spectra of the a DBR measured at 15° incidence for s- (black) and p-polarized (blue) light. Both polarizations exhibit similar spectral features with high reflectivity in the stopband region around 480 nm. The inset shows the image of as-prepared 7.5 pairs DBR. (b) Calculated peak reflectivity of an ideal quarter-wave DBR as a function of the number of layer pairs N, using $R_{\max} = \left(\frac{1-\left(\frac{n_L}{n_H}\right)^{2N}}{1+\left(\frac{n_L}{n_H}\right)^{2N}} \right)^2$, with refractive indices $n_L = 1.35$ and $n_H = 1.85$, corresponding to the low- and high-index layers used in the present TiOH:PVA/Nafion DBRs. The calculation shows that the reflectivity approaches a plateau after approximately seven pairs, consistent with the experimentally measured reflectivity of the fabricated DBRs.

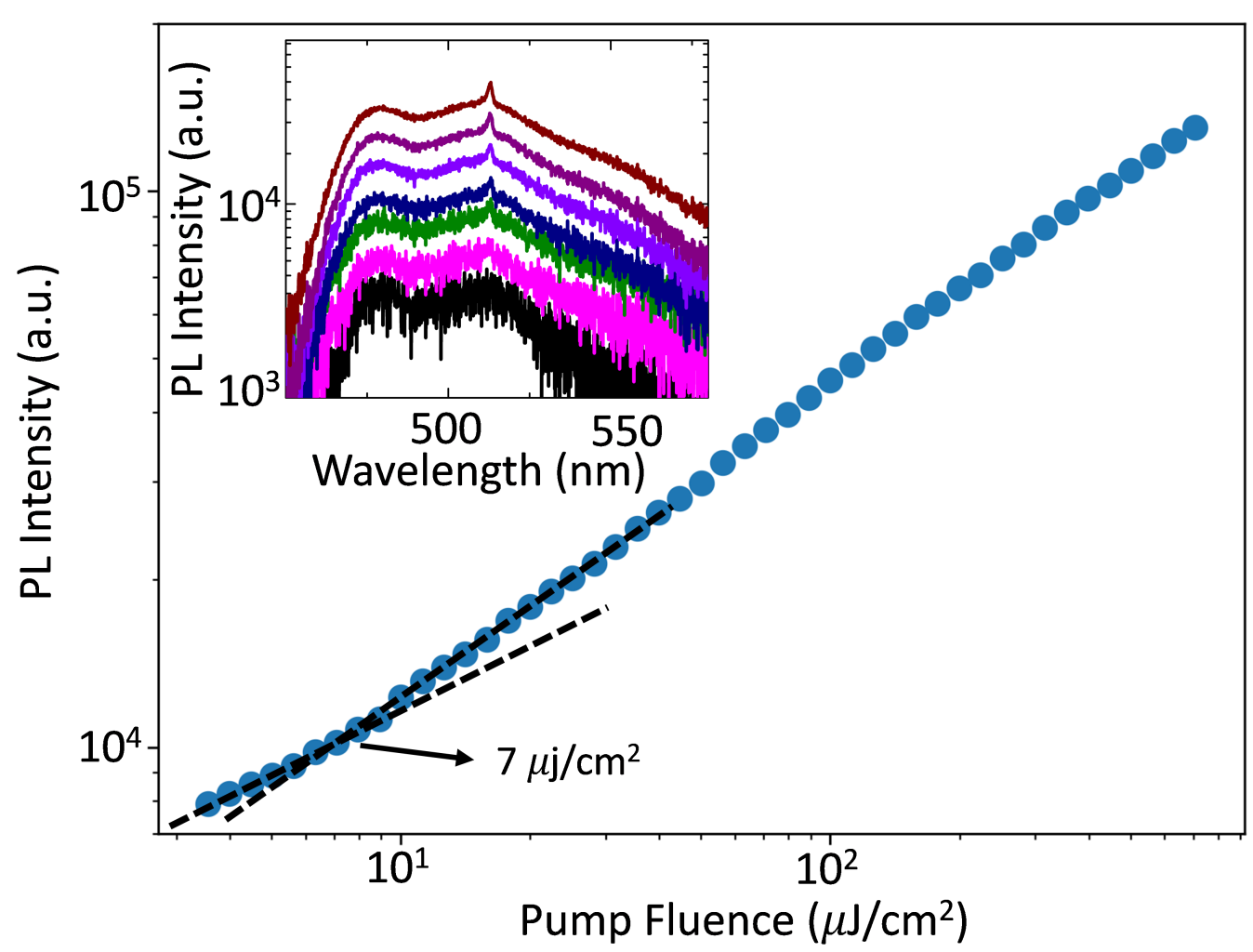

Supplementary Fig. 2. Power-dependent photoluminescence (PL) measurements of the bare film. The main plot shows the integrated PL intensity as a function of pump fluence, exhibiting a nearly linear increase without a clear threshold behavior. The inset presents the corresponding PL spectra at different fluences, showing amplified spontaneous emission (ASE) with increasing pump power.

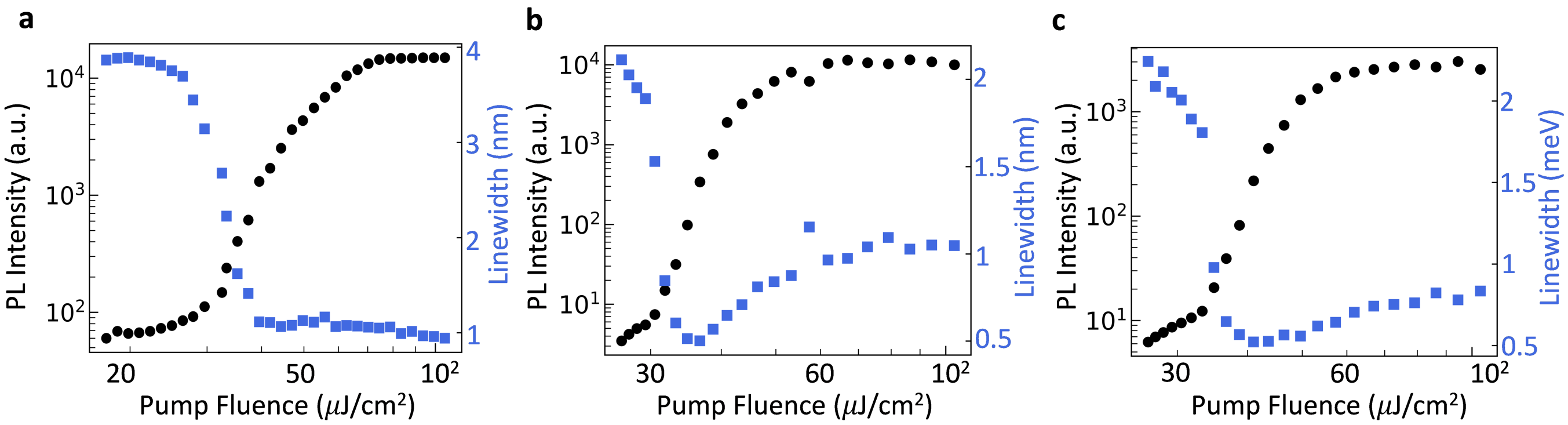

Supplementary Fig. 3. Power-dependent measurements showing the integrated PL intensity (black dots) and emission linewidth (blue squares) as a function of pump fluence for different pump–collection polarization configurations: (a) s-polarized pump and p-polarized collection, (b) p-polarized pump and p-polarized collection, and (c) p-polarized pump and s-polarized collection. All configurations exhibit a clear threshold-like increase in PL intensity accompanied by linewidth narrowing, characteristic of a polariton lasing. No significant differences in threshold and linewidth are observed between polarization configurations, indicating negligible cavity anisotropy or birefringence.

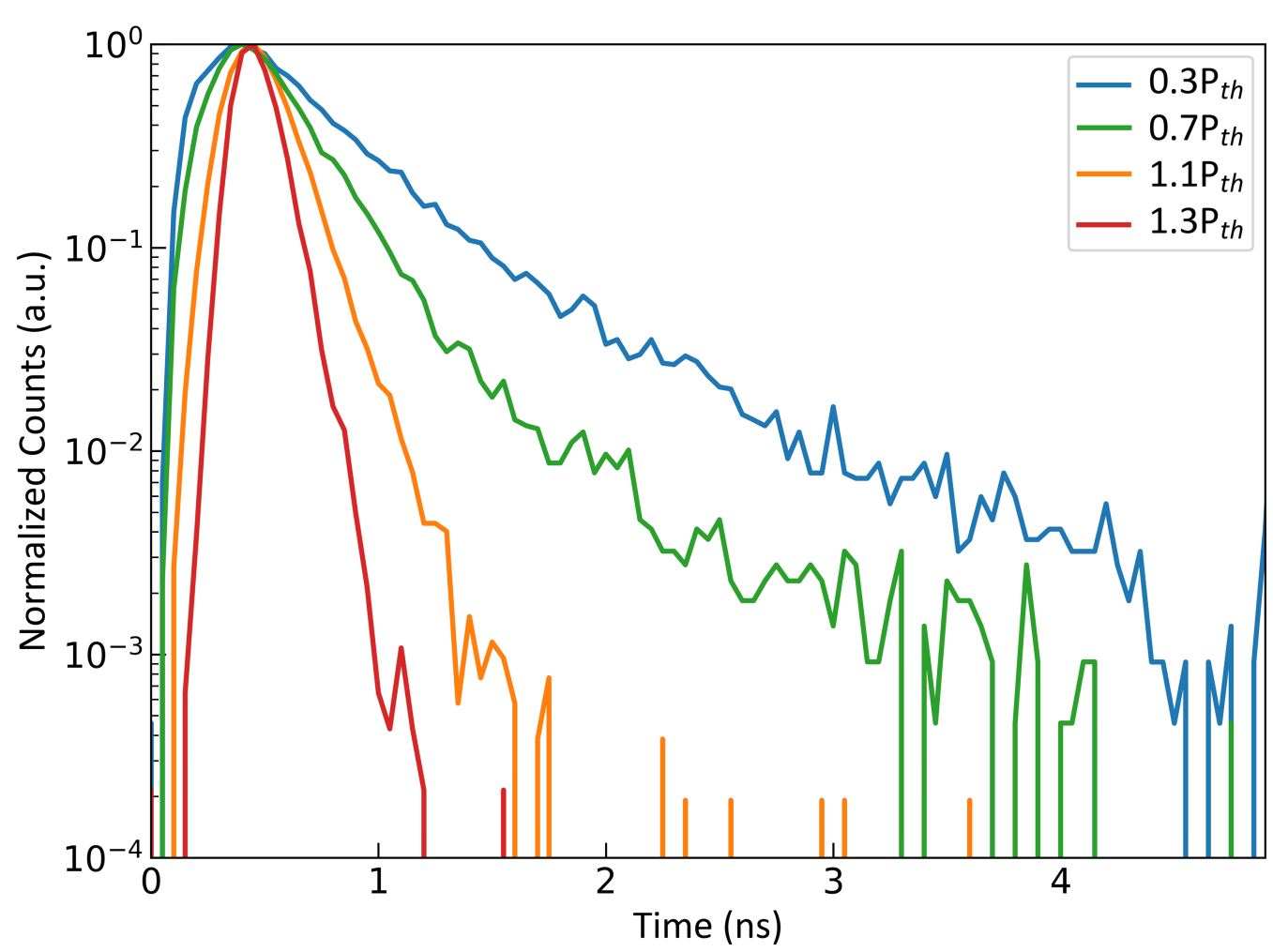

Supplementary Fig. 4. Time-resolved photoluminescence (TRPL) decay curves of the microcavity emission recorded at different excitation powers. As the pump fluence increases from 0.3 Pth to 1.3 Pth, the decay time shortens significantly indicating accelerated relaxation dynamics as the system transitions to the lasing regime.

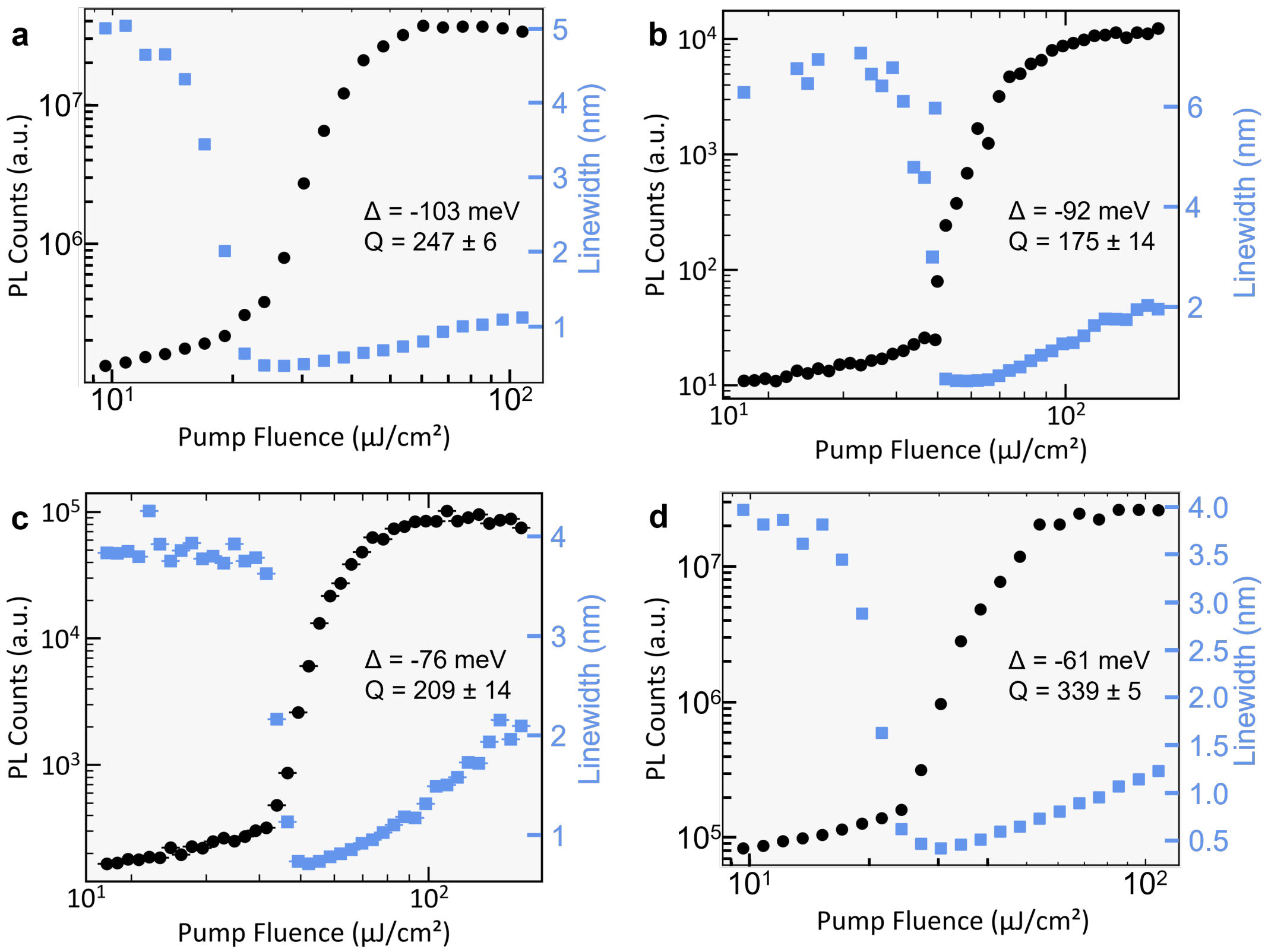

Supplementary Fig. 5. Power-dependent PL characteristics with emission peaks at (a) 511.25 nm, (b) 510 nm, (c) 508 nm, and (d) 506.25 nm. Panels (a - d) show the integrated PL intensity (black) and linewidth (blue) as functions of pump fluence, exhibiting a clear threshold-like behavior and linewidth narrowing, indicative of the transition to the polariton lasing regime. Δ is the exciton-cavity detuning and Q is determined from five low-fluence measurements integrated over small collection angles. The data in panels (a) and (d) are from the same measurements as the data in Supplementary Figs. 11 and 12 of the cavity detunings Δ = -103 meV and Δ = -61 meV, respectively.

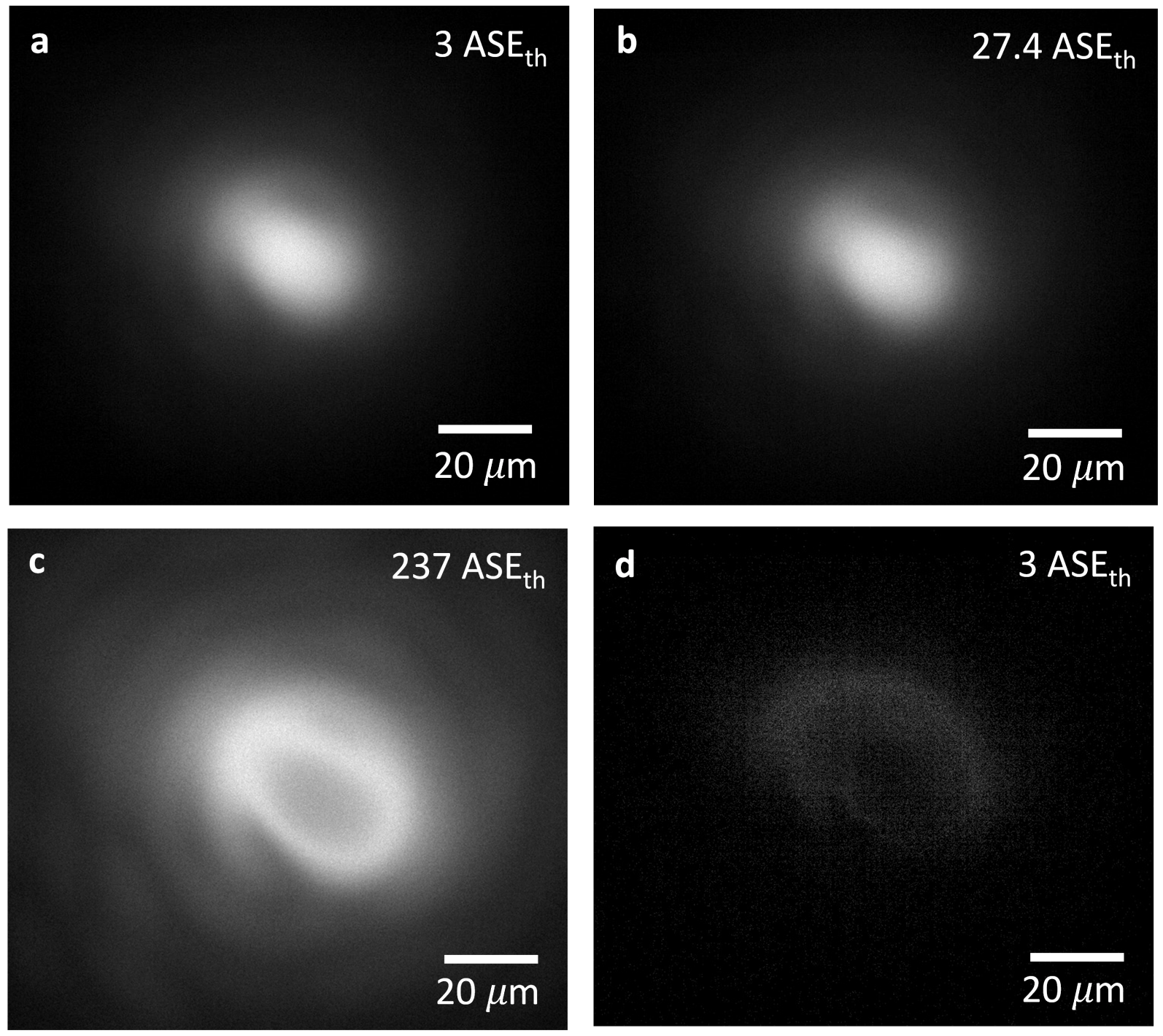

Supplementary Fig. 6. Real-space emission images of a neat DPAVB:PS film recorded during a deliberately overpumped ASE cycling experiment performed at a repetition rate of 200 kHz. Images were recorded at absorbed pump fluences corresponding to (a) 3 $\mathrm{ASE_{th}}$ (21 $\mu\mathrm{J\,cm^{-2}}$), (b) 27.4 $\mathrm{ASE_{th}}$ (192 $\mu\mathrm{J\,cm^{-2}}$), and (c) 237 $\mathrm{ASE_{th}}$ (1659 $\mu\mathrm{J\,cm^{-2}}$). Panel (d) shows the same spot after reducing the absorbed pump fluence back to the initial low-fluence condition used in panel (a), namely 3 $\mathrm{ASE_{th}}$ (21 $\mu\mathrm{J\,cm^{-2}}$), following exposure to the highest fluence. The strongly reduced emission intensity and modified spatial profile in (d) indicate irreversible photoinduced damage of the neat film under high-fluence excitation. This permanent damage behavior is distinct from the reversible annular emission observed in the polariton microcavities.

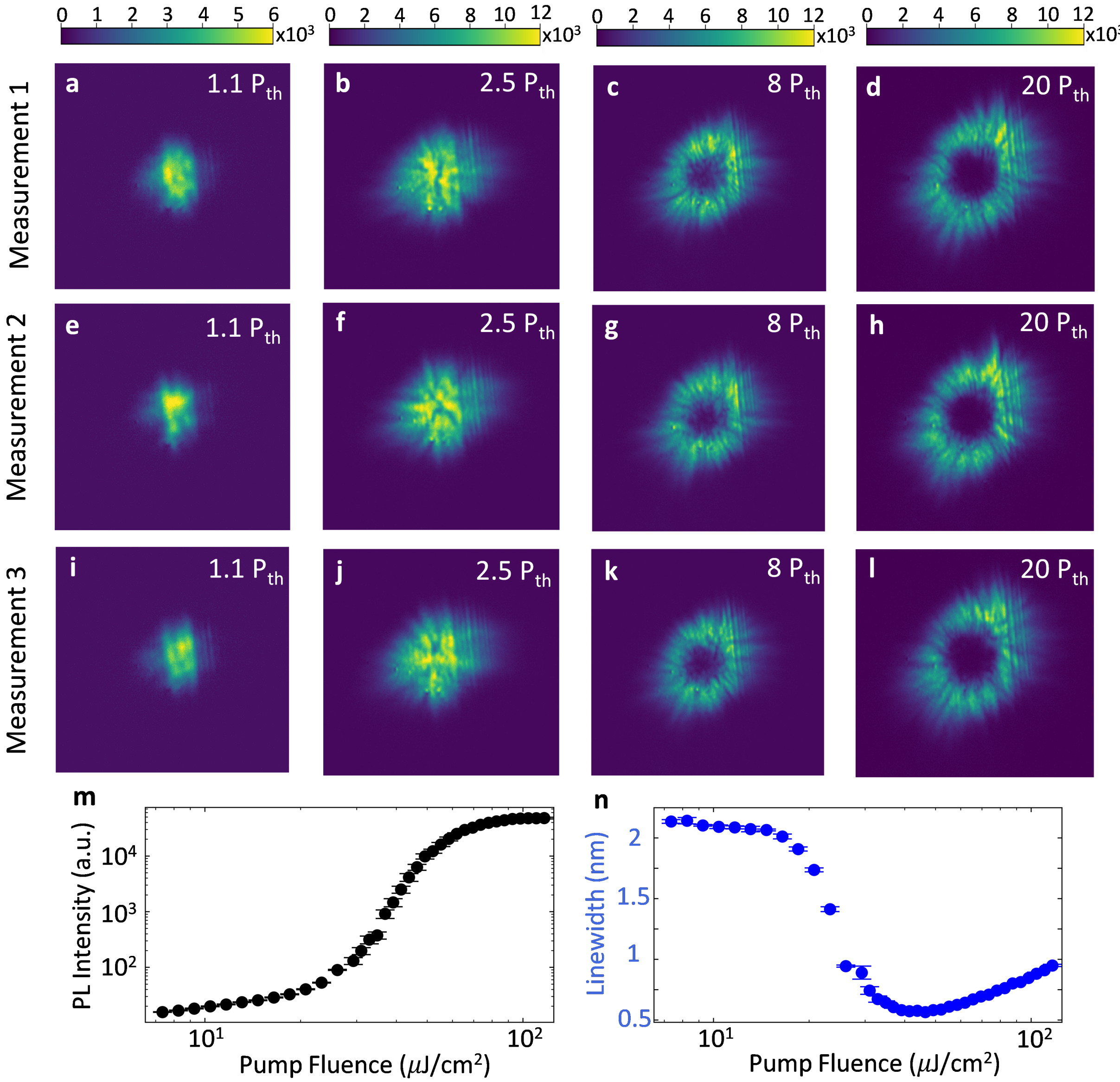

Supplementary Fig. 7. (a-l)Real-space emission images recorded at the same spot under different excitation powers and repeated three times to verify reproducibility. The measurement order is (a)→(b)→(c)→(d)→(e)→(f)→(g)→(h)→(i)→(j)→(k)→(l). At low excitation (1.1 P_{th}), localized emission is observed, while at higher powers (8 P_{th}) and (20 P_{th}), a ring-shaped emission pattern consistently appears in all measurements. The repeatable emergence of the ring structure confirms that it is an intrinsic feature of the condensate rather than damage to the film. (m,n) Power-dependent LP photoluminescence intensity (black dots) and corresponding LP linewidth (blue dots). Error bars represent the standard deviation obtained from three independent measurements. The measurements were performed in the same sample spot back to back and the power curves were also done back to back after full power cycles.

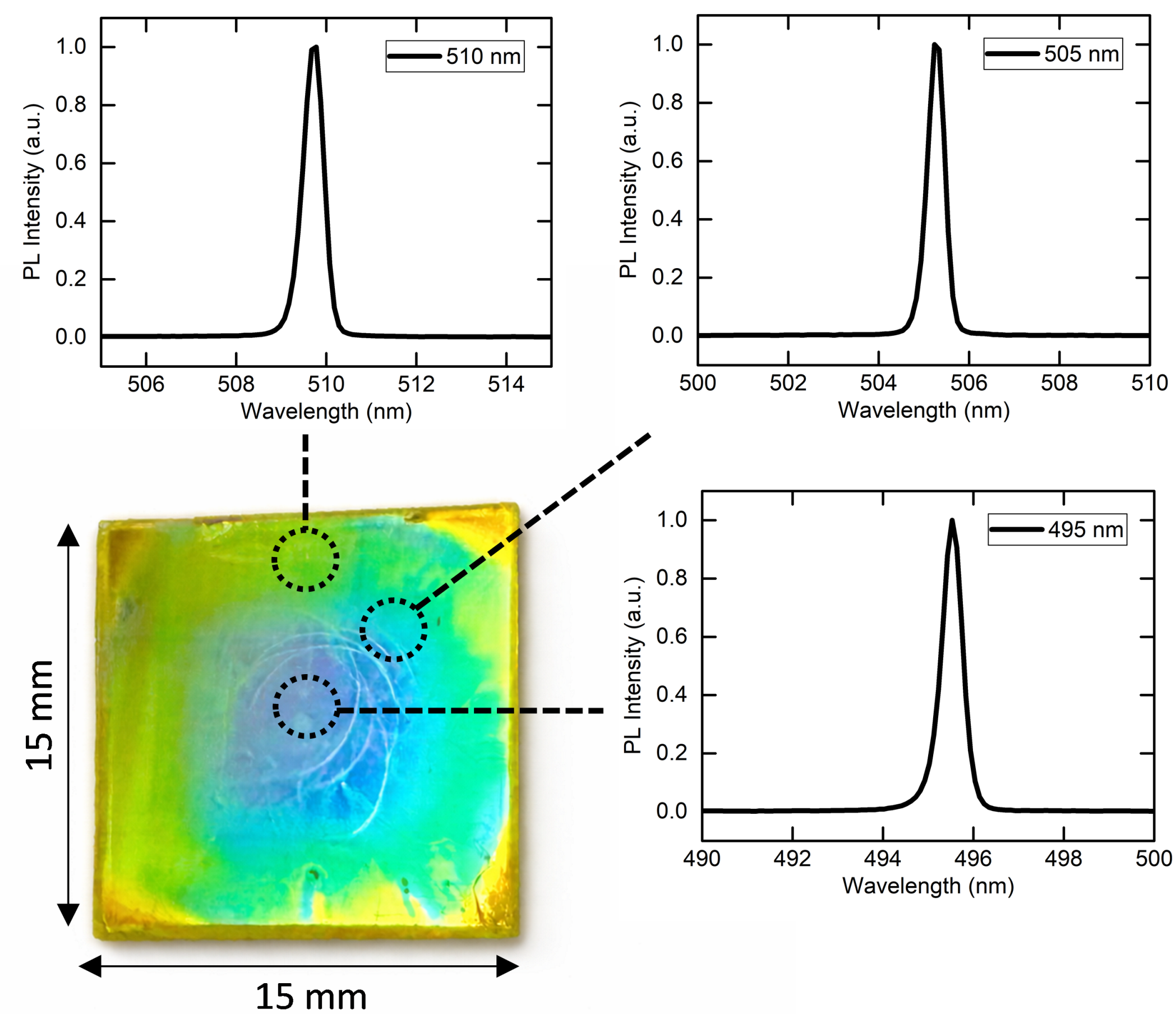

Supplementary Fig. 8. Photograph of the fabricated microcavity sample illustrating the spatial thickness gradient induced by spin coating. The inherent coating dynamics produce a gradual cavity thickness variation across the substrate, resulting in position-dependent detuning. By translating the collection area across the sample (dashed lines), different detuning conditions can be accessed on a single device. Representative PL spectra recorded at three distinct spatial positions (dotted circles) show clear spectral shifts (495 nm, 505 nm, and 510 nm), directly demonstrating the spatial variation of the cavity resonance. Even though the detuning changes notably in position, the Q values and the lasing thresholds vary only slightly (330-240 and 20-23 μJ cm^{-2}, respectively, see Fig. 3a and Supplementary Figs. 5a and 5d indicating that the cavity quality remains uniformly high across the sampled positions of the coated device.

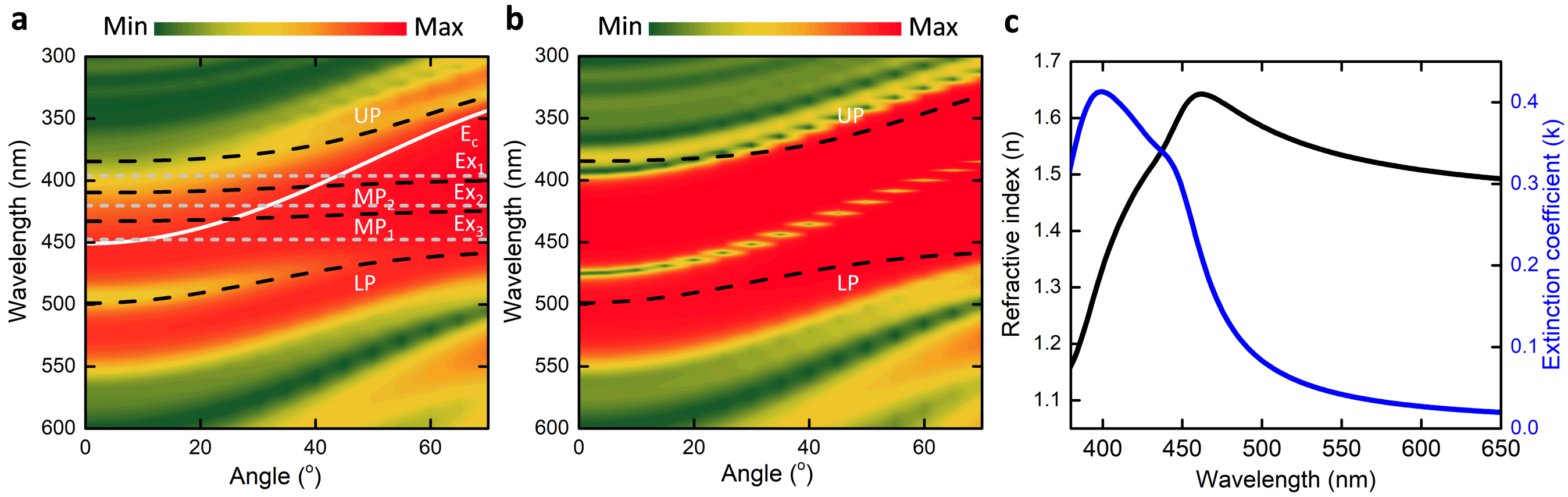

Supplementary Fig. 9. Transfer-matrix modelling (TMM) of the cavity reflectivity using the experimentally measure refractive index (n and k). (a) Full-cavity TMM including excitonic resonances, where the coupling parameters are taken from the CHOM fitted to the experimental reflectivity data. The simulation reproduces the observed anticrossing and dispersion curvature, consistent with sustained strong coupling. Dashed lines indicate the calculated polariton branches obtained from the coupled oscillator model (CHO). (b) TMM of the empty cavity without excitonic resonances, showing only the bare cavity and higher-order Bragg modes. In this case, no anticrossing or mode splitting is observed. Dashed lines mark the dispersion of the bare cavity photon mode for reference. (c) Experimentally measured complex refractive index (n and k) of the DPAVB:PS film.

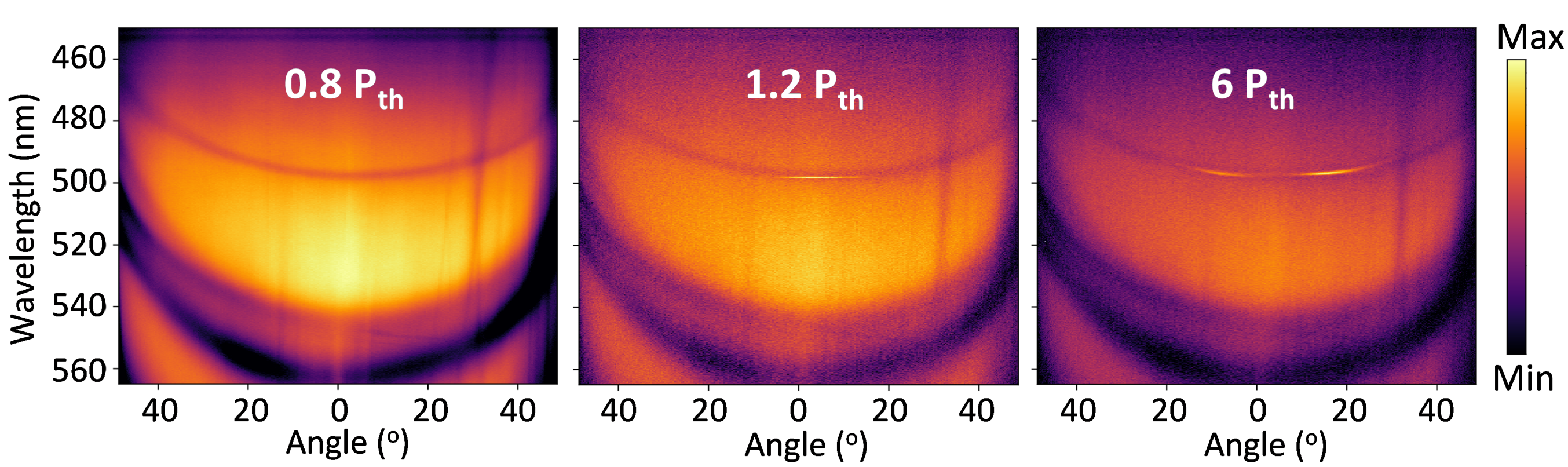

Supplementary Fig. 10. Steady-state angle-resolved reflection measured below and above threshold at $0.8\,P_{\mathrm{th}}$, $1.2\,P_{\mathrm{th}}$, and $6\,P_{\mathrm{th}}$. The LP dispersion remains spectrally and angularly unchanged across threshold, with no observable distortion or shift of the LP or higher-order Bragg modes. Above threshold, the lasing emission remains strongly confined within the established LP branch and does not evolve toward the bare cavity photon dispersion.

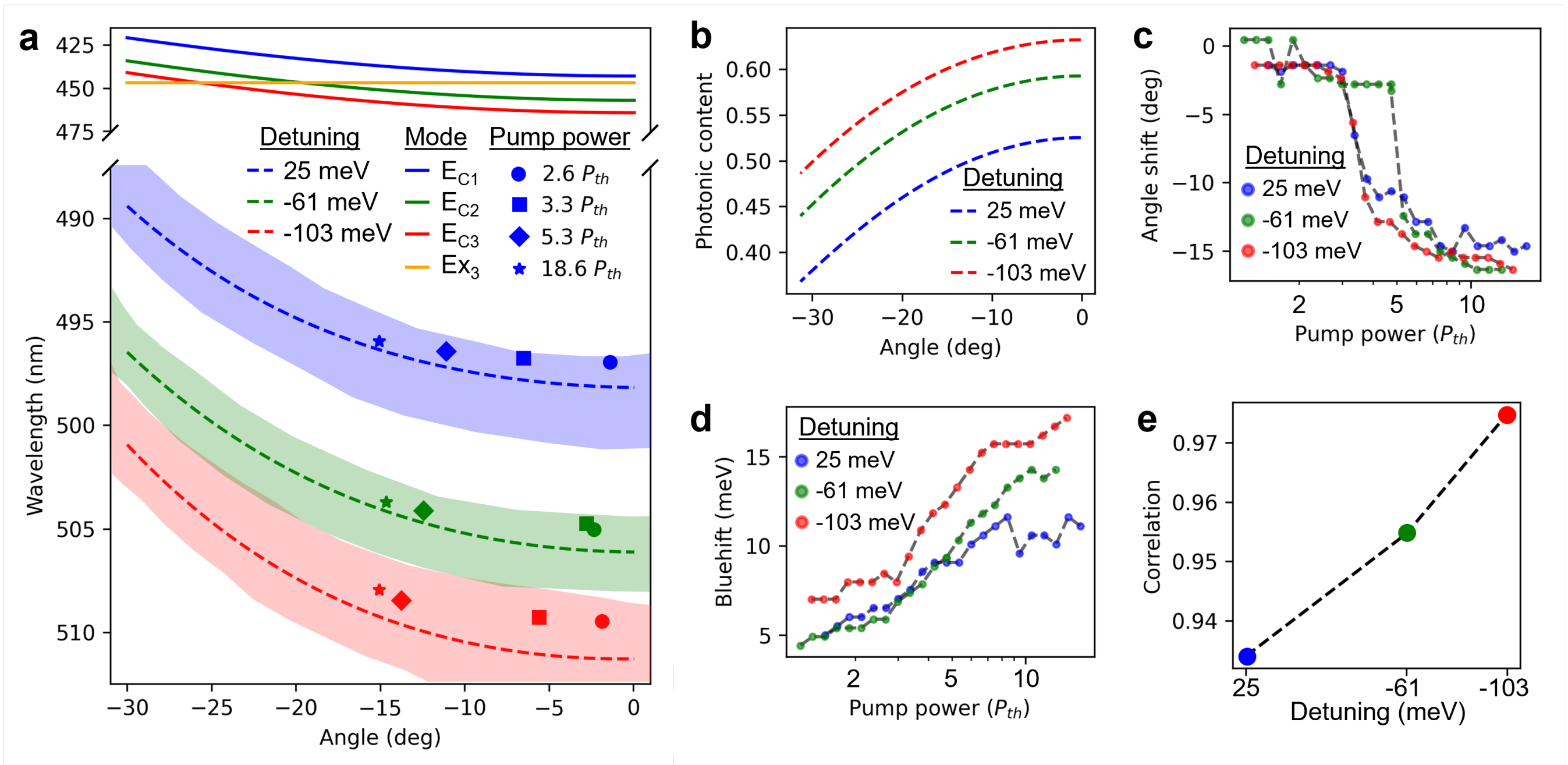

Supplementary Fig. 11. The blueshift and angle shift along the below-threshold LP dispersion for three differently detuned cavities. (a) Above-threshold emission obeys the below-threshold dispersion. The solid lines are the exciton energy E_{X_3} and the dispersions of the bare cavities (E_{C1}, E_{C2}, and E_{C3}). The dashed lines show the below-threshold dispersions of the LP branches corresponding to the matching-colored cavity dispersions. The shaded areas highlight the parameter regions where the below-threshold EL intensity is at least half of its maximum. The different scatter markers show the highest emission intensity angle-wavelength coordinates at different pump powers, where $P_{\rm th}$ is the lasing threshold. While the pump power increases, the emission undergoes blueshift, but simultaneously the emission angle shifts keeping the highest intensity clearly within the below-threshold dispersion's linewidth. (b) The coupled harmonic oscillator model simulation of the Hopfield coefficients shows that increasing the detuning makes the polariton more photonic. (c) The angle shift and (d) the blueshift of the highest emission coordinate as function of the pump power. (e) The Spearman ρ correlation coefficient between the blueshift and angle shift for the three differently detuned cavities from (c-d). The correlation is normalized between -1 and +1, where -1 means perfect anticorrelation, 0 means uncorrelated properties, and +1 means perfect correlation. Here the correlation increases monotonically with detuning, suggesting stronger dependence between blueshift and dispersion for the more photonic polaritons. The integrated PL intensity and linewidth for the detunings 25 meV, -61 meV, and -103 meV, are shown in Figs. 3a, and 5c and d, respectively. The integrated PL intensities over the full excitation range are shown in Supplementary Fig. 12e.

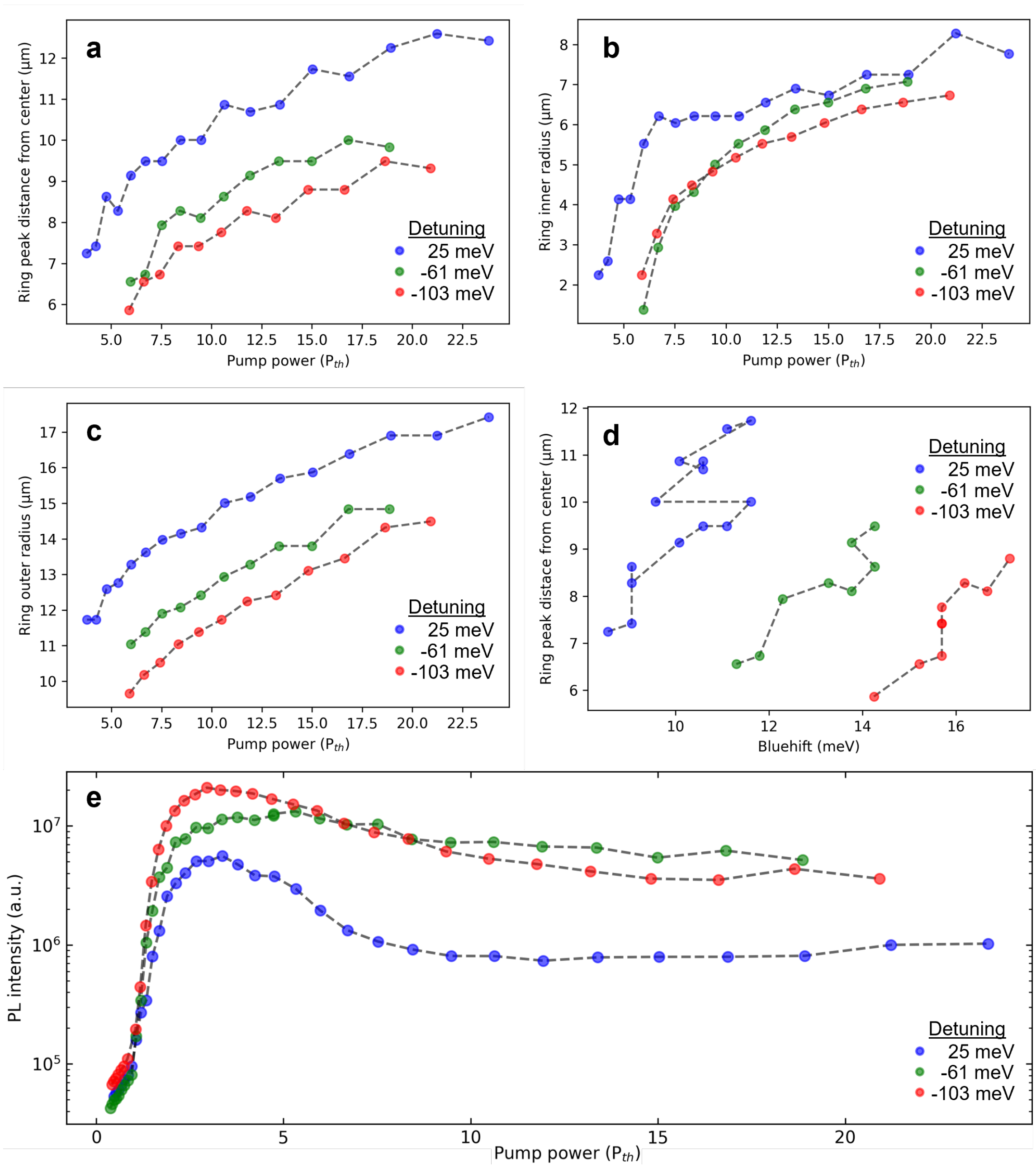

Supplementary Fig. 12. The real-space emission ring. As the pump power increases beyond the lasing threshold, a ring-like feature emerges around the pump spot's center in the real-space emission. Further increasing the pump power enlarges the ring as seen in Fig. 4 of the main article. For each detuning and pump power, the 2D real-space intensity distribution is averaged over all the angles around the pump center position and the resulting radial intensity distribution is analyzed in panels (a-c). (a) Ring's maximum intensity shift, (b) inner half-maximum shift, and (c) the outer half-maximum shift as function of the pump power. The maximum intensity and outer radius shifts are larger for the less-detuned cases which correspond to higher excitonic polariton content. (d) The ring peak's distance from the pump center plotted against the corresponding blueshift. This real-space data for each detuning was measured simultaneously with the corresponding k-space data analyzed in Supplementary Fig. 11. (e) The integrated PL intensities over the full excitation range used in this and Supplementary Fig. 11

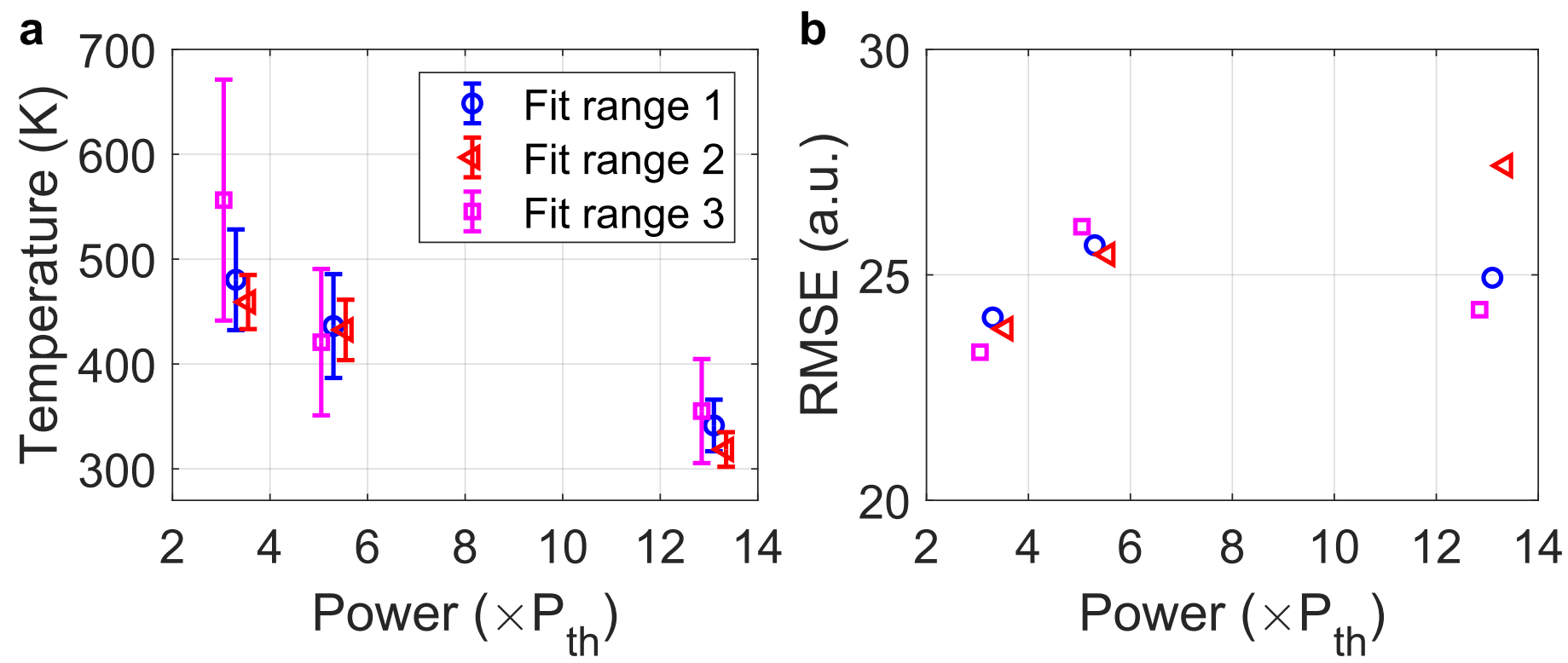

Supplementary Fig. 13. Fit sensitivity analysis. (a) Temperatures obtained from Maxwell–Boltzmann fits over different spectral ranges: Fit range 1 (blue circles) corresponds to the 50 meV window used in the manuscript Fig. 5; Fit range 2 (red triangles) uses a wider 70 meV window; and Fit range 3 (magenta squares) uses a narrower 30 meV window. The wider and narrower windows are expanded or reduced symmetrically around the fitting range indicated in manuscript Fig. 5. The error bars indicate 95% confidence bounds. (b) Root-mean-squared error (RMSE) of each fit shown in (a); the values are reported in the same (arbitrary) intensity units as the spectra in Fig. 5(d–f), enabling direct comparison. For clarity, the data points in both panels are offset by ±0.25 units along the x-axis to resolve overlapping markers.

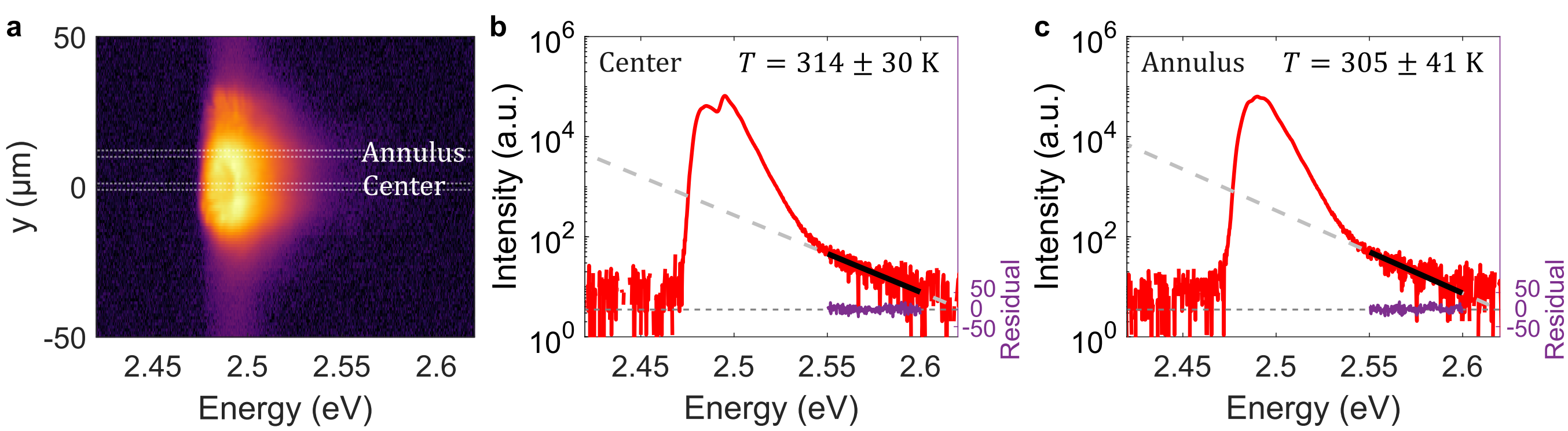

Supplementary Fig. 14. Spatial dependence of fit temperature. Comparison of the fitted thermal tails in different spatial regions (center vs. annulus). (a) Real-space-resolved emission spectrum for the pump $13.1P_{\mathrm{th}}$, corresponding to manuscript Fig. 5c. (b–c) Fits of the thermal tail to the Maxwell–Boltzmann distribution for the (b) center and (c) annulus regions, with the extracted fit temperatures and their 95% confidence intervals. Fit residuals are plotted in linear scale on the right-hand y-axis. The line spectra in panels (b–c) are obtained by integrating over the respective regions indicated in (a) within a ±3 µm range.
