# Supplementary material for: A fully solution-processed organic microcavity laser in the strong light-matter coupling regime
Source: Nat Commun. 2026 Jul 3;17:8280. doi: 10.1038/s41467-026-75118-1 (PMC13469614; doi:10.1038/s41467-026-75118-1)
Supplement: Supplementary file 3 [file 41467_2026_75118_MOESM3_ESM.pdf]

nature portfolio

Peer Review File

A fully solution-processed organic microcavity laser in the strong light–matter coupling regime

Corresponding Author: Professor Konstantinos Daskalakis

This manuscript has been previously reviewed at another journal. This document only contains information relating to versions considered at Nature Communications.

This file contains all reviewer reports in order by version, followed by all author rebuttals in order by version.

Attachments originally included by the reviewers as part of their assessment can be found at the end of this file.

Version 0:

Reviewer comments:

Reviewer #1

(Remarks to the Author)

I have carefully examined the revised manuscript, the revised Supplementary Information, and the authors' rebuttal letter. In my view, the authors have responded constructively and substantially to the main concerns raised in the previous review round. The revised submission is significantly strengthened in both evidentiary support and clarity of interpretation. I therefore recommend acceptance after minor corrections.

The main advance of the work is now articulated more clearly than in the original version: not simply the use of spin coating as a fabrication route, but the realization of a fully solution-processed organic microcavity platform that exhibits lasing in the strong-coupling regime, together with an interesting high-density redistribution dynamics of the condensate. In the revised form, this combination is sufficiently novel and potentially relevant for the readership of Nature Communications.

The strongest improvement is the way the authors now support the strong-coupling interpretation. The original concerns regarding the visibility of the polariton branches, the ambiguity of the coupled-oscillator fitting, and the possibility of weak-coupling/photon-lasing behaviour have been addressed seriously. In particular, the addition of the transfer-matrix-based comparison using measured optical constants, together with the empty-cavity comparison, materially strengthens the claim that the observed anti-crossing is not an artefact of an over-parameterized coupled-oscillator model. Likewise, the new above-threshold reflectivity data and the detuning-dependent analyses considerably improve the case that the emission remains confined to the lower-polariton branch rather than asymptotically approaching the bare cavity mode.

I also find the added discussion of sample provenance, spatial detuning variation, and reproducibility helpful. The revised manuscript and Supplementary Information now make it much clearer which datasets were acquired from which detunings/spatial positions and why this is possible within the same coated sample. The additional reporting of (Q)-factors, fitting uncertainties, and fabrication conditions is appropriate and improves transparency.

The revision is also stronger in its treatment of the interaction-driven annular redistribution. The authors have appropriately softened the earlier wording that seemed to attribute the effect exclusively to polariton-polariton interactions. The revised language now acknowledges the likely role of both polariton-polariton and polariton-reservoir interactions, which is more accurate and better supported by the data. The new quantitative ring analysis across detunings is useful and makes the phenomenology more convincing. Similarly, the reversibility discussion is much improved, and the comparison with irreversible bare-film behavior helps exclude a simple damage-based explanation.

The thermal-tail analysis is also in a better state. The authors now explain more carefully why the Maxwell-Boltzmann fit is restricted to the high-energy tail, and they appropriately reinterpret the extracted temperature as a phenomenological slope parameter rather than as proof of full thermal equilibrium. The sensitivity and spatial-dependence analyses in the Supplementary Information are valuable additions and sufficiently address my earlier concern on over-interpretation.

Overall, the revised manuscript is now much more balanced. Several earlier claims have been made more precise, the evidentiary chain is stronger, and the conclusions are now better matched to what the data actually show.

That said, I still have a few minor corrections:

The manuscript should be given a final pass to remove wording that remains slightly overstated in places. In particular, statements implying that the fabrication route “addresses” the intrinsic instability of organic semiconductors still read too strongly. The revised discussion already moves in the right direction, but the final clean version should avoid implying that solution processing solves the stability problem itself; rather, it provides a pragmatic and experimentally accessible route compatible with such material constraints.

The Summary/Abstract should continue to be phrased carefully with respect to scalability and technological outlook. Phrases suggesting a direct route to scalable quantum photonic technologies are somewhat broader than what is experimentally demonstrated here. The present study convincingly establishes an important proof of principle and a viable fabrication platform; it does not yet establish device scalability in the engineering sense. A slightly more measured formulation would strengthen the paper.

The terminology around polariton lasing, polariton condensation, and microcavity laser has improved, but the final accepted version should ensure that the terminology is fully clean and consistent throughout. In the tracked-changes version, some sentences remain awkward because of overlaid edits. The final production version should be checked carefully so that these definitions read smoothly and unambiguously.

I encourage the authors to ensure that the final clean manuscript explicitly preserves the now-improved caveat that the high-density outward redistribution is consistent with interaction-driven dynamics but does not by itself uniquely identify the microscopic relative weights of polariton-polariton and polariton-reservoir contributions.

In conclusion, I find that the authors have addressed the substantive scientific concerns sufficiently. The revised manuscript is stronger, clearer, and more convincing, and the remaining issues are minor and editorial in nature. I do not wish to re-review the manuscript.

Reviewer #3

(Remarks to the Author)

[Editorial Note: See attachment at end of file]

Reviewer #4

(Remarks to the Author)
The authors report the realization of a fully solution-processed Fabry–Pérot-type organic microcavity incorporating an organic semiconductor active layer, and demonstrate polariton condensation within this platform. The work addresses an important practical challenge in organic polaritonics, where high-reflectance dielectric mirrors are typically fabricated using vacuum deposition or sputtering processes. By implementing a fully solution-processed approach, the authors aim to provide a potentially more cost-effective and scalable route toward organic microcavity devices. Furthermore, the revised manuscript significantly strengthens the evidence for polariton condensation. The inclusion of TMM modelling, additional experimental data, and more detailed analysis collectively provides a more comprehensive and convincing dataset.
The demonstration of a robust, fully solution-processed platform is of interest and may provide useful guidance for future device design and fabrication strategies. However, given the broad scope of Nature Communications and the fact that fully spin-coating-processed microcavities have already been reported in previous studies (e.g., Adv. Optical Mater. 2017, 5, 1700523; Nanoscale, 2019, 11, 8978), it would be important to more clearly demonstrate the physical property part, for example, why novel physical insights like ring-shaped condensation are uniquely enabled by this fabrication approach.
I provide the following comments for the authors’ consideration.

Comment 1
The manuscript shows a ring-shaped emission profile at high excitation densities. It is not clear why this feature has not been observed in previous studies, and why it appears in the present fully spin-coated microcavity.
One possibility is that higher excitation densities can be reached in this system, for example due to lower threshold or better stability compared to conventional cavities, leading to higher polariton densities.
It would be helpful if the authors could clarify this point. A comparison with microcavities fabricated by conventional methods (such as sputtering) may also help to understand the origin of this behaviour.

Comment 2
Considering that exciton diffusion typically occurs via intermolecular interactions, a neat film of the active material would be ideal. However, in this study, the dye is dispersed in a PS matrix.
It should be interesting to know whether increasing the dye concentration, or approaching a neat-film-like condition, would affect the observed ring-shaped redistribution, such as its spatial extent or dynamics.

Comment 3
What polariton density is needed to induce the ring-shaped diffusion? Would it be more appropriate to define the threshold in terms of the polariton population rather than the pump power?

Comment 4
In this study, the excitation power can be increased to more than ten times the threshold. Is there any transition from polariton lasing to photon lasing under such conditions? For example, is there any features of a two-threshold behavior?

Comment 5
The manuscript mentions scalability, but it is not clear what device size can realistically be achieved with the present approach.
Since spin coating typically has limitations in terms of large-area uniformity, it would be helpful if the authors could comment on the scalability of this method. For example, could similar structures be fabricated using other solution-based techniques such as blade coating?

Comment 6
In typical microcavity structures for polariton formation, one side of the DBR is often designed to have a reflectivity close to 100%. In this study, however, the top DBR consists of 7.5 pairs with a reflectivity of about 94%.
It is not clear why this design was chosen. Would increasing the number of DBR pairs and achieving higher reflectivity improve the cavity performance or polariton properties?

Comment 7
The advantage of using polymer-based materials is their flexibility; is this microcavity device actually flexible?

Version 1:

Reviewer comments:

Reviewer #3

(Remarks to the Author)

[Editorial Note: See attachment at end of file]

Open Access This Peer Review File is licensed under a Creative Commons Attribution 4.0 International License, which permits use, sharing, adaptation, distribution and reproduction in any medium or format, as long as you give appropriate credit to the original author(s) and the source, provide a link to the Creative Commons license, and indicate if changes were made.
In cases where reviewers are anonymous, credit should be given to 'Anonymous Referee' and the source.

The images or other third party material in this Peer Review File are included in the article's Creative Commons license, unless indicated otherwise in a credit line to the material. If material is not included in the article's Creative Commons license and your intended use is not permitted by statutory regulation or exceeds the permitted use, you will need to obtain permission directly from the copyright holder.
To view a copy of this license, visit https://creativecommons.org/licenses/by/4.0/

Response to the Reviewer reports

A fully solution-processed organic microcavity laser in the strong light–matter coupling regime

Hassan A. Qureshi, Henri Lyyra, Akseli Korkeamäki, Oskar Tuomi, Antti J. Moilanen, and Konstantinos S. Daskalakis
Corresponding author: konstantinos.daskalakis@utu.fi

We thank the Editors and Reviewers for their time, care, and constructive assessment of our manuscript. We are very grateful for the thoughtful comments provided in this round of review, which have helped us further improve the clarity, balance, and precision of the manuscript. In response, we have carefully revised the text, refined several interpretations and forward-looking statements, corrected unclear wording, and added targeted clarifications where appropriate.

Below, we provide a point-by-point response to all comments raised in this round. In the revised manuscript, deleted text is shown as ~~red strikethrough~~, and new text is shown in blue.

Reviewer #1 (Remarks to the Authors): *I have carefully examined the revised manuscript, the revised Supplementary Information, and the authors' rebuttal letter. In my view, the authors have responded constructively and substantially to the main concerns raised in the previous review round. The revised submission is significantly strengthened in both evidentiary support and clarity of interpretation. I therefore recommend acceptance after minor corrections.*

The main advance of the work is now articulated more clearly than in the original version: not simply the use of spin coating as a fabrication route, but the realization of a fully solution-processed organic microcavity platform that exhibits lasing in the strong-coupling regime, together with an interesting high-density redistribution dynamics of the condensate. In the revised form, this combination is sufficiently novel and potentially relevant for the readership of Nature Communications.

The strongest improvement is the way the authors now support the strong-coupling interpretation. The original concerns regarding the visibility of the polariton branches, the ambiguity of the coupled-oscillator fitting, and the possibility of weak-coupling/photon-lasing behaviour have been addressed seriously. In particular, the addition of the transfer-matrix-based comparison using measured optical constants, together with the empty-cavity comparison, materially strengthens the claim that the observed anti-crossing is not an artefact of an over-parameterized coupled-oscillator model. Likewise, the new above-threshold reflectivity data and the detuning-dependent analyses considerably improve the case that the emission remains confined to the lower-polariton branch rather than asymptotically approaching the bare cavity mode.

I also find the added discussion of sample provenance, spatial detuning variation, and reproducibility helpful. The revised manuscript and Supplementary Information now make it much clearer which datasets were acquired from which detunings/spatial positions and why this is possible within the same coated sample. The additional reporting of (Q)-factors, fitting uncertainties, and fabrication conditions is appropriate and improves transparency.

The revision is also stronger in its treatment of the interaction-driven annular redistribution. The authors have appropriately softened the earlier wording that seemed to attribute the effect exclusively to polariton-polariton interactions. The revised language now acknowledges the likely role of both polariton-polariton and polariton-reservoir interactions, which is more accurate and better supported by the data. The new quantitative ring analysis across detunings is useful and makes the phenomenology more convincing. Similarly, the reversibility discussion is much improved, and the comparison with irreversible bare-film behavior helps exclude a simple damage-based explanation.

The thermal-tail analysis is also in a better state. The authors now explain more carefully why the Maxwell-Boltzmann fit is restricted to the high-energy tail, and they appropriately reinterpret the extracted temperature as a phenomenological slope parameter rather than as proof of full thermal equilibrium. The sensitivity and spatial-dependence analyses in the Supplementary Information are valuable additions and sufficiently address my earlier concern on over-interpretation.

Overall, the revised manuscript is now much more balanced. Several earlier claims have been made more precise, the evidentiary chain is stronger, and the conclusions are now better matched to what the data actually show.

That said, I still have a few minor corrections:

Authors' response

We sincerely thank the Reviewer for this very positive and motivating feedback, and for the careful and constructive assessment of our revised manuscript. We are delighted that the Reviewer finds the revised submission significantly strengthened in both evidentiary support and clarity of interpretation.

We would also like to thank the Reviewer once again for the insightful suggestions provided in the previous review round. These comments encouraged us to study our samples in greater depth, which proved extremely valuable for improving our understanding of the system and for strengthening the manuscript accordingly. The additional experiments, analyses, and clarifications that were carried out in response to those comments have, in our view, substantially improved the quality, rigor, and balance of the work.

We are grateful for the Reviewer's constructive guidance throughout this process, which has played an important role in improving the manuscript.

Minor Comment 1

The manuscript should be given a final pass to remove wording that remains slightly overstated in places. In particular, statements implying that the fabrication route "addresses" the intrinsic instability of organic semiconductors still read too strongly. The revised discussion already moves in the right direction, but the final clean version should avoid implying that solution processing solves the stability problem itself; rather, it provides a pragmatic and experimentally accessible route compatible with such material constraints.

Answer

Upon re-reading the manuscript, we agree that the original wording was too strong. We have therefore revised the corresponding sentence in the Summary to avoid implying that the fabrication route itself solves the intrinsic instability of organic semiconductors. Specifically, the sentence was changed from "... ~~addresses a fundamental challenge of organic semiconductors: their intrinsic instability~~" to "... provides a pragmatic and experimentally accessible route compatible with the intrinsic material constraints of organic semiconductors." We have thoroughly reviewed the manuscript once more and modified the statements pointed out by the reviewer (see also below). In our assessment, we did not find any further statements that appear over-stated.

Minor Comment 2

The Summary/Abstract should continue to be phrased carefully with respect to scalability and technological outlook. Phrases suggesting a direct route to scalable quantum photonic technologies are somewhat broader than what is experimentally demonstrated here. The present study convincingly establishes an important proof of principle and a viable fabrication platform; it does not yet establish device scalability in the engineering sense. A slightly more measured formulation would strengthen the paper.

Answer
We thank the reviewer for bringing this up. Our intention was to provide a forward-looking perspective, but we appreciate that this should be phrased more carefully. We have therefore revised the relevant sentences to frame the present work more appropriately as a proof-of-principle and a viable experimental platform for future developments, rather than as a direct demonstration of device scalability in the engineering sense. In the Abstract, the sentence was changed from "... ~~establish a route toward scalable polaritonic and quantum photonic technologies~~ ..." to "... establish a viable platform for exploring future polaritonic and quantum photonic technologies ...". In the Summary, the sentence was changed from "... ~~provide a foundation for scalable classical and quantum photonic applications~~ ..." to "... provide a promising platform for future classical and quantum photonic applications ...".

Minor Comment 3
The terminology around polariton lasing, polariton condensation, and microcavity laser has improved, but the final accepted version should ensure that the terminology is fully clean and consistent throughout. In the tracked-changes version, some sentences remain awkward because of overlaid edits. The final production version should be checked carefully so that these definitions read smoothly and unambiguously.

Answer
We thank the Reviewer for this helpful observation. We have now made a final pass through the manuscript to ensure that the terminology is used consistently and reads smoothly throughout. In particular, we use "polariton lasing" as the primary term for the observed above-threshold emission, while "polariton condensation" is retained where it refers more specifically to the condensate state or to broader literature usage. Below is a list of revised sentences: (i) In the Summary opening sentence, we replaced "... first fully solution-processed ~~polariton microcavity laser~~ ..." with "... first fully solution-processed organic polariton microcavity to exhibit polariton lasing ..."; (ii) We revised "polariton condensation pathway" to a smoother formulation, which now reads from "... our platform has revealed a new ~~polariton condensation pathway~~ ..." to "... our platform has revealed a new redistribution pathway of the polariton condensate ...".

Minor Comment 4
I encourage the authors to ensure that the final clean manuscript explicitly preserves the now-improved caveat that the high-density outward redistribution is consistent with interaction-driven dynamics but does not by itself uniquely identify the microscopic relative weights of polariton-polariton and polariton-reservoir contributions.

Answer
We agree that the caveat should be stated more explicitly in the final clean manuscript. We have therefore revised the relevant discussion in the main text to make this point explicit. Below is a list of revised sentences: (i) "This spatially resolved behaviour, together with the momentum redistribution observed in Fig. 3, is consistent with repulsive ~~polariton–polariton and polariton–reservoir interactions driving the condensate outward, adding to the emission intensity at the edges of the pumped region~~" was revised to "This spatially resolved behaviour, together with the momentum redistribution observed in Fig. 3, is consistent with repulsive interaction-driven outward dynamics of the condensate, to which both polariton–polariton and polariton–reservoir interactions, while not distinguishing their relative roles or excluding additional contributions from saturation-, migration-, or transport-related mechanisms (Betzold et al., ACS Photonics (2019), 7, 384–392; Yagafarov et al., Commun. Phys. (2020), 3, 18; Berghuis et. al., ACS Nano, vol. 18, no. 46, pp. 31987–31994, (2024)". (ii) The sentence "This outward redistribution ~~not only reflects repulsive~~

~~polariton interactions but also provides~~ a mechanism to avoid exciton saturation at the pump center..." was revised to "This outward redistribution may provide a mechanism to avoid exciton saturation at the pump center...". (iii) In the Figure 4 caption, we modified "The central emission saturates at high fluence, while off-center regions brighten with progressively higher thresholds ~~, consistent with repulsive polariton - reservoir and polariton–polariton interactions driving outward flow~~." (iv) We removed the following sentence from the caption of Supplementary figure S12: "~~This suggests that the hole forms due to the repulsive polariton-polariton interaction, which increases with the polariton's excitonic content.~~"

Reviewer #1 (Final remarks to the Authors): *In conclusion, I find that the authors have addressed the substantive scientific concerns sufficiently. The revised manuscript is stronger, clearer, and more convincing, and the remaining issues are minor and editorial in nature. I do not wish to re-review the manuscript.*

Authors' response
We sincerely thank the Reviewer for the time, care, and constructive guidance provided throughout both rounds of review. The Reviewer's comments have been invaluable in helping us improve the quality, clarity, and balance of the manuscript. We are particularly grateful for the Reviewer's positive final assessment and for supporting publication of our work in Nature Communications.

Reviewer #3 (Remarks to the Authors): *Qureshi et al. report an organic exciton polariton laser microcavities fabricated entirely by solution processing, which includes both organic molecules and DBR prepare by solution method. And reversible, interaction driven redistribution of polariton lasing is observed at a high excitation density, revealing significant polariton laser behavior in organic microcavities. However, I don't believe that the all-solution device manufacturing method and the flowable exciton polariton laser are highly innovative. Therefore, I don't recommend this work publishing in Nature Communications. It is more suitable for other professional journals.*

Authors' response
We thank the Reviewer for this clear assessment and for articulating the concern regarding the level of novelty and general significance of the work. We respectfully disagree with the view that the present manuscript is insufficiently innovative for publication in Nature Communications.

In our view, the significance of the manuscript lies not in either element considered separately, but in their combination. First, the work establishes a fully solution-processed organic microcavity platform that reaches lasing in the strong-coupling regime. Second, this platform can be driven reliably to high excitation densities, where we observe a reversible redistribution of the polariton condensate. We believe this gives the manuscript significance beyond fabrication alone, by combining a methodological advance with physically interesting high-density condensate dynamics relevant to specialists in organic polaritonics and photonics.

We also emphasize that the manuscript was already revised in the previous round to make this combined significance clearer, rather than presenting the contribution as fabrication novelty alone. In the present revision, we have further refined the manuscript in response to the current reports by (i) softening forward-looking wording where necessary so as not to overstate scalability or technological readiness, (ii) improving the consistency and precision of the terminology used for polariton lasing, condensation, and microcavity operation, (iii) further clarifying the interpretation of the high-density redistribution so that it is presented as being consistent with repulsive interaction-driven dynamics without over-interpreting the relative roles of polariton–polariton and polariton–reservoir contributions, while also not excluding possible contributions from saturation-, migration-, or transport-related mechanisms, and (iv) adding targeted clarifications and supporting

material regarding DBR uniformity, and the absence of a second threshold indicative of photon lasing. The corresponding manuscript changes are detailed in the relevant point-by-point responses in this letter.

We therefore respectfully submit that the manuscript is of interest not only because it demonstrates a fully solution-processed, strongly coupled organic microcavity, but also because it couples this methodological advance to a physically interesting high-density condensate redistribution phenomenon. In our view, this combination of an experimentally accessible and scalable fabrication route with new insights into nonlinear polariton dynamics makes the work relevant not only to specialists in organic polaritonics and photonics, but also to the broader community working on photonic materials, light-matter coupling, and coherent emitters. We therefore maintain that the manuscript is appropriate for Nature Communications.

Comment 1

The author claims that one of the innovations of this manuscript is the preparation of organic films and DBR through a full-solution method, but no relevant data is provided on the uniformity of DBR, which makes it difficult for readers to believe that large-area, high reflectivity DBR can be obtained through a simple spin-coating method. The author should provide a cross-sectional view of the thickness of DBR within the microscopic region, such as scanning electron microscope (SEM) imaging, to confirm the thickness and uniformity of each layer of DBR medium throughout the entire size range.

Answer

We thank the Reviewer for this helpful comment. We agree that the manuscript should be phrased carefully so as not to imply that the present study demonstrates engineering-scale uniformity or large-area manufacturability. Our intention is instead to establish a fully solution-processed microcavity platform that is experimentally accessible and that may support future development of larger-area solution-processed photonic structures. We also note that, in the present revision, we have softened broader forward-looking wording where necessary so as not to overstate present large-area manufacturability, while keeping the focus on the experimentally accessible nature of the platform.

At the same time, we would also like to clarify that the manuscript already contains several pieces of evidence relevant to the structural and optical uniformity of the DBR-based cavity. First, the cross-sectional SEM image in Fig. 1a shows the full multilayer cavity structure with well-defined alternating layers and good interfacial integrity. Second, Supplementary Fig. S8 presents a photograph of the fabricated sample together with spectra acquired from different positions across the coated area, demonstrating a smooth position-dependent detuning arising from the expected spin-coating thickness gradient rather than irregular cavity failure. Third, as noted in the revised caption of Supplementary Fig. S8, the cavity quality factor and lasing threshold vary only slightly across the sampled positions: Supplementary Fig. S5 demonstrates clear polariton lasing behavior and high cavity quality at several different detunings/positions.

To make this point clearer to the reader, we have revised the manuscript text and Supplementary Information as follows. In the main text, the sentence "This approach produces mirrors with high reflectivity ($\sim$ 94%, Supplementary Fig. S1) and excellent interfacial integrity ~~(right side of Fig. 1a)~~, resulting in optical cavities with a quality factor (Q) $>$ 325 ..." was revised to "This approach produces mirrors with high reflectivity ($\sim$ 94%, Supplementary Fig. S1) and excellent interfacial integrity with well-defined multilayer formation visible in the cross-sectional SEM image in Fig. 1a, resulting in optical cavities with a quality factor (Q) $>$ 325 ...". In the caption of Supplementary Fig. S8, we extended the text to include "indicating that the cavity quality remains uniformly high across the sampled positions of the coated device.". We also added the sample dimensions along

the x- and y-axes to Supplementary Fig. S8 to make the spatial extent of the coated area more explicit.

More broadly, we note that solution-processed DBRs are of significant current interest as a photonic materials platform, as discussed both in our recent article on planar solution-based DBRs (Advanced Materials Interfaces, vol. 10, no. 19, p. 2202206, (2023)) and in a recent Advanced Materials Perspective (Advanced Materials vol. 38, e07028 (2026)) on titanium oxide hydrate/polyalcohol optical materials. We cite these works only to provide context for why such platforms are of broader interest; however, the central point here is that the present manuscript already provides direct structural and optical evidence that the fabricated microcavities are sufficiently high quality and uniform to support strong coupling and polariton lasing across different positions on the sample.

Comment 2

The angle-dependent reflectivity in Figure 2b is the most important data to demonstrate strong exciton-photon coupling. The author claims to have seen the LPB, MPB and UPB. However, the MPB are not seen in the Figure 2b, and the author needs to provide more intuitive and clear evidence. In addition, how are the three exciton peak positions of DPAVB determined? The author needs to provide the corresponding absorption spectra of pure DPAVB films with the same thickness as in Figure 2b.

Answer

We agree that the angle-dependent reflectivity is central to demonstrating strong exciton–photon coupling. For this reason, in addition to the color map in Fig. 2b, we also present the angle-resolved spectra in Fig. 2c, where the individual branches are more clearly resolved over selected angular ranges, especially above 50°. We agree that this was not made sufficiently clear in the original manuscript, and we now address this by adding the following text to the caption: Note that all four polariton branches are most clearly visible in the angle-resolved spectra for angles exceeding 50°. For the Reviewer's convenience, we also include response-letter spectra at 55° and 60°, where the LP, MP1, MP2, and UP branches can be identified directly.

Regarding the assignment of the excitonic resonances, we would like to clarify that these were determined from the measured optical response of the actual active layer used in the cavity, namely DPAVB dispersed in a PS matrix. As already shown in Fig. 2a, the manuscript includes the absorption spectrum of DPAVB:PS together with the cavity reflectivity and emission spectra. The excitonic energies used in the coupled-oscillator description were assigned from the features in this measured absorption spectrum, and were cross-checked against the experimentally measured complex refractive index (n, k) of the DPAVB:PS film shown in Supplementary Fig. S9c. These assignments are also consistent with previous reports on DPAVB-based polariton microcavities (McGhee et. al., J. Mater. Chem. C (2022), 10, 4187–4195).

We respectfully note that the relevant absorption reference for the present strong-coupling analysis is the DPAVB:PS active layer actually used in the cavity, rather than a neat DPAVB film of the same thickness, because the polariton dispersion is determined by the excitonic resonances and optical constants of the material system embedded in the microcavity.

Finally, to clarify the origin of the excitonic energies, we revised the text around Fig. 2a from "Figure 2a shows the normal-incidence reflectivity of the polariton microcavity together with the absorption, photoluminescence, and ASE spectra of DPAVB in a PS matrix." to "Figure 2a shows the normal-incidence reflectivity of the polariton microcavity together with the absorption, photoluminescence, and ASE spectra of DPAVB in a PS matrix, from which the excitonic resonances used in the coupled-oscillator analysis are assigned, supported by the measured optical constants in Supplementary Fig. S9c."

We hope this clarification and the revised figure presentation address the Reviewer's concern.

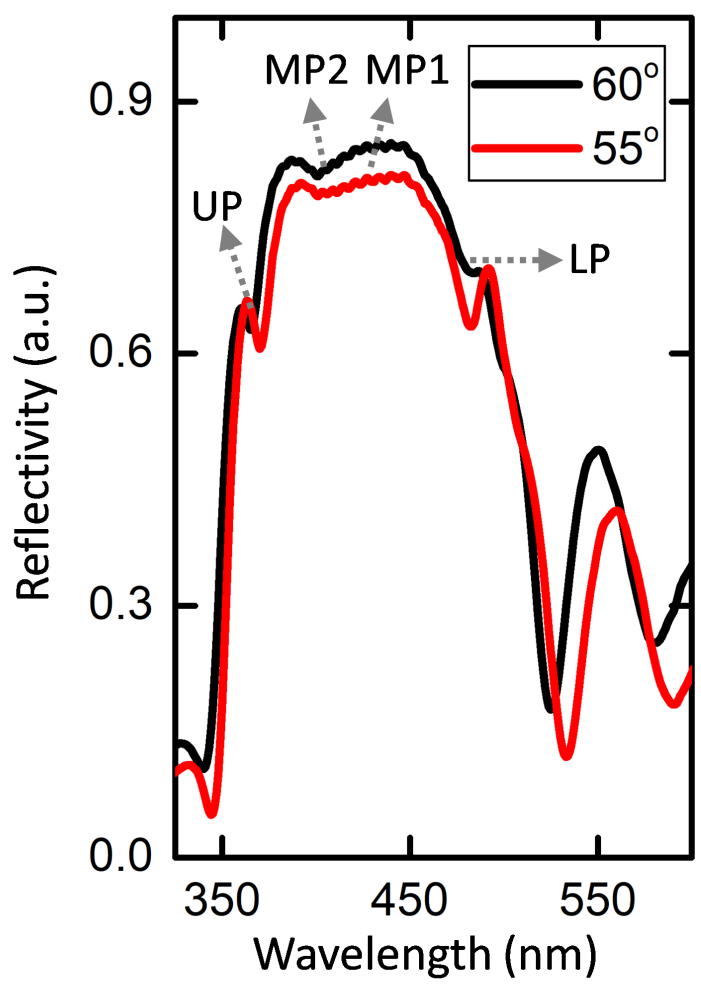

Fig. R1: Reflectivity spectra of the DPAVB:PS polariton microcavity measured at 55° (red) and 60° (black), highlighting the spectral positions of the upper polariton (UP), middle polariton branches (MP1 and MP2), and lower polariton (LP).

Comment 3

Figure 4 shows that after increasing the pump density, the condensate redistributes outward as it gains momentum, forming an annular emission profile due to the repulsion of polariton-reservoir and polariton-polariton interactions. However, according to previous reports, the blue-shift mechanism of polariton energy in organic semiconductor microcavities is different from that in inorganic semiconductor microcavities. The energy blue-shift in organic semiconductor microcavities is due to the interplay of the saturation effects and intermolecular energy migration, or the phase space filling effects, rather than the interaction between polaritons (Comm. Phys. 2020, 3(1), 18; ACS Photon. 2019, 7(2), 384-392). The author should reconsider the physical mechanism of redistribution of polariton towards higher energy.

Answer

We thank the Reviewer for this important comment. We agree that the microscopic origin of blueshifts in organic microcavities should be discussed with particular care. Indeed, in organic semiconductor microcavities, energy blueshifts can arise from phase-space-filling effects—most notably saturation of the electronic transition—as well as from intermolecular energy-migration processes, both of which can produce effective interaction signatures without requiring direct polariton–polariton interactions. We therefore appreciate the Reviewer's suggestion and have expanded the discussion accordingly. In particular, we have retained the citation to Yagafarov et al., Commun. Phys. (2020), 3, 18, and added the relevant earlier work of Betzold et al., ACS Photonics (2019), 7, 384–392. We have also included a recent report on organic polaritons in bound states in the continuum in nanoparticle arrays, where outward-flow / annular emission under Gaussian pumping was discussed in terms of lifetime-limited ballistic transport and associated loss channels (ACS Nano (2024), 18, 31987–31994). This provides an additional and potentially relevant perspective on possible mechanisms for combined energy shift and outward redistribution in organic polariton systems.

Regarding the present work, we would like to emphasize that the phenomenon discussed here is not only a simple energy blueshift. Experimentally, we observe correlated evolution in energy, momentum, and real space. In addition, as shown in Supplementary Figs. S11 and S12, the energy shift, momentum redistribution, and real-space annular profile all depend on the excitonic content of the lower polariton. We view this combined detuning dependence as qualitatively supportive of an interaction- and reservoir-mediated outward-redistribution picture. To further clarify this point, we have added a new panel in Supplementary Fig. S12e showing the integrated photoluminescence

intensity over the full excitation range for the different detunings. Importantly, no second nonlinear threshold is observed, which would be expected for a transition to weak-coupling / photon lasing. At the same time, we agree that these observations do not by themselves uniquely exclude contributions from intermolecular energy migration, ballistic propagation, or combinations of these mechanisms.

Moreover, our above-threshold reflectivity measurements show that the emission remains confined to the lower-polariton branch and does not evolve toward the bare cavity mode (Supplementary Fig. S10). This is important because, as shown in our previous theoretical work on organic microcavity polariton lasing (Arnardottir et al., Phys. Rev. Lett. 125, 233603 (2020)), saturation of the emitters' electronic transition can reduce the Rabi splitting, shifting the polariton dispersion toward the uncoupled cavity-photon mode. In the present experiments, this behaviour is not observed. We therefore conclude that phase-space-filling-induced dispersion renormalization cannot account for the high-density redistribution reported here.

Having said this, our interpretation does not exclude that saturation-, migration-, or transport-related mechanisms may also contribute. A detailed microscopic understanding will require substantial further theoretical and experimental investigation, which lies beyond the scope of the present work but is planned for future study. Our intention is therefore not to claim that the observed high-density behaviour is uniquely explained by direct polariton–polariton interactions alone. Rather, we interpret the data more cautiously as being consistent with repulsive interaction-driven outward dynamics, with likely contributions from both the reservoir and the polariton population, while not resolving their relative microscopic weights.

We have therefore reconsidered and softened the wording in the manuscript and updated the relevant literature accordingly, as detailed below.

- In the main text, the sentence "This spatially resolved behaviour, together with the momentum redistribution observed in Fig. 3, is consistent with repulsive ~~polariton–polariton and polariton–reservoir interactions driving the condensate outward, adding to the emission intensity at the edges of the pumped region~~" was revised to "This spatially resolved behaviour, together with the momentum redistribution observed in Fig. 3, is consistent with interaction-driven outward dynamics of the condensate, to which both polariton–polariton and polariton–reservoir interactions, while not distinguishing their relative roles or excluding additional contributions from saturation-, migration-, or transport-related mechanisms (Betzold et al., ACS Photonics (2019), 7, 384–392; Yagafarov et al., Commun. Phys. (2020), 3, 18; Berghuis et. al., ACS Nano, vol. 18, no. 46, pp. 31987–31994, (2024)."

- In the Figure 4 caption, we modified "The central emission saturates at high fluence, while off-center regions brighten with progressively higher thresholds ~~, consistent with repulsive polariton - reservoir and polariton–polariton interactions driving outward flow~~."

- We also revised the following sentence from "This outward redistribution ~~not only reflects repulsive polariton interactions but also provides~~ a mechanism to avoid exciton saturation at the pump center, thereby protecting against degradation of strong coupling." to "This outward redistribution may provide a mechanism to avoid exciton saturation at the pump center, thereby protecting against degradation of strong coupling."

- In addition, in the Summary we softened the corresponding wording from "Although a comprehensive study of this novel physical mechanism is planned, we ~~attribute this to repulsive polariton–polariton and polariton-reservoir interactions that depopulate the pumped center and feed the periphery~~ ..." to "Although a comprehensive study of this novel physical mechanism is planned, we interpret the observed redistribution as being consistent with repulsive interaction-driven dynamics that depopulate the pumped center and feed the periphery ..."

- We removed the following sentence from the caption of Supplementary figure S12: "~~This suggests that the hole forms due to the repulsive polariton-polariton interaction, which increases with the polariton's excitonic content.~~"

We hope this revised wording better reflects the current level of mechanistic certainty.

Comment 4

On page 11 of the manuscript, it is described as "As shown in Supplementary Fig. S6, neat DPAVB:PS films exhibit irreversible damage under ASE cycling, whereas the polariton microcavities can be repeatedly driven across threshold without any change in emission profile or intensity (Supplementary Fig. S7)". I don't understand why the optical stability of DPAVB films in microcavities is enhanced. As far as I know, organic semiconductors are generally unstable, especially after high-power pump density irradiation, their stability decreases and is accompanied by an increase in the condensation threshold. However, the condensation data in Figure S7 is surprisingly stable. Can the author explain why the optical properties of the film are so stable?

Answer

We thank the Reviewer for this careful comment, which made us realize that our original presentation of Supplementary Fig. S6 and the corresponding discussion could indeed give a misleading impression.

We identified that, due to a production error, the legend and caption of Supplementary Fig. S6 contained incorrect information regarding the threshold-referenced excitation values. In fact, the irreversible damage observed in the neat DPAVB:PS films occurs only when the films are pumped at fluences far above the ASE threshold, namely up to 37 $\mathrm{ASE_{th}}$ (262 μJ/cm^2). We would also like to clarify, and apologize for not stating this explicitly in the previous version, that the bare-film damage experiment was deliberately performed under much harsher excitation conditions than the microcavity cycling experiment. In Supplementary Fig. S6 the repetition rate was increased from 2 kHz, used for the microcavity measurement in Supplementary Fig. S7, to 200 kHz in order to make the damage signature clearly visible. Supplementary Fig. S6 should therefore not be interpreted as a quantitative stability comparison between the neat film and the microcavity. We have corrected Supplementary Fig. S6 accordingly and also changed the Supplementary Fig. S6 caption from "~~Real-space emission images of the film recorded at 0.5 P_{th}, P_{th}, and 7 P_{th}, respectively, show the progressive formation of a ring-like feature as the pump power increases. (d) Image of the same spot taken again at 0.5 P_{th} after exposure to higher power (7 P_{th}) clearly shows the same ring pattern, confirming that the observed ring in the film originates from irreversible damage compared to the reversible ring shape emission observed in polariton microcavities.~~" to "Real-space emission images of a neat DPAVB:PS film recorded during a deliberately overpumped ASE cycling experiment performed at a repetition rate of 200 kHz. Images were recorded at absorbed pump fluences corresponding to (a) 3 $\mathrm{ASE_{th}}$ (21 $\mu\mathrm{J\,cm^{-2}}$), (b) 27.4 $\mathrm{ASE_{th}}$ (192 $\mu\mathrm{J\,cm^{-2}}$), and (c) 237 $\mathrm{ASE_{th}}$ (1659 $\mu\mathrm{J\,cm^{-2}}$). Panel (d) shows the same spot after reducing the absorbed pump fluence back to the initial low-fluence condition used in panel (a), namely 3 $\mathrm{ASE_{th}}$ (21 $\mu\mathrm{J\,cm^{-2}}$), following exposure to the highest fluence. The strongly reduced emission intensity and modified spatial profile in (d) indicate irreversible photoinduced damage of the neat film under high-fluence excitation. This permanent damage behavior is distinct from the reversible annular emission observed in the polariton microcavities.".

We have also updated Supplementary Fig. S7 to include a pump-fluence cycling measurement of the polariton microcavity up to $20P_{\mathrm{th}}$, corresponding to the highest excitation range used for the annular-emission measurements reported in this work, as shown in the new panel (e) of Supplementary Fig. S12. Upon reducing the pump fluence back to the initial value, the emission profile and intensity recover fully, confirming that the annular redistribution observed under these conditions is reversible and not associated with permanent optical damage.

Our conclusion is therefore not that the microcavity intrinsically stabilizes DPAVB:PS. Rather, the updated cycling measurements show that, over the excitation range used for the annular-emission

data reported here, the microcavity emission remains reproducible and does not exhibit the irreversible damage signature observed in the deliberately overpumped bare DPAVB:PS blend film. Thus, the annular redistribution observed in the polariton lasing measurements is reversible under the present experimental conditions and cannot be attributed to permanent optical damage.

To make this clearer, we revised the sentence in the manuscript from "~~As shown in Supplementary Fig. S6, neat DPAVB:PS films exhibit irreversible damage under ASE cycling, whereas the polariton microcavities can be repeatedly driven across threshold without any change in emission profile or intensity (Supplementary Fig. S7). This confirms that the observed spatial profiles are reversible—contrasting with the irreversible rings observed in bare films—and therefore intrinsic to the condensate rather than arising from material or optical damage~~" to "Supplementary Fig. S6 shows a control experiment illustrating the appearance of irreversible optical damage in a neat DPAVB:PS film. By contrast, no such behaviour is observed in the polariton microcavities over the excitation range where annular emission emerges (Supplementary Fig. S7), confirming that the observed annular redistribution is reversible and not a damage-induced artifact.

We hope that the corrected Supplementary Fig. S6, the new panels added to Supplementary Figs. S7 and S12, and the revised wording now make this point clear, and we thank the Reviewer again for bringing this issue to our attention.

Reviewer #3 (Final remarks to the Authors): *Overall, although the work in this manuscript is interesting, its innovation is insufficient, and the physical mechanism of the redistribution of polariton energy remains to be discussed. So I don't recommend publishing it in Nature Communications.*

Authors' response

We thank the Reviewer for the careful assessment of our work. We respectfully disagree with this overall conclusion, but hope that the revisions and detailed point-by-point responses above have addressed the Reviewer's concerns.

Reviewer #4 (Remarks to the Authors): *The authors report the realization of a fully solution-processed Fabry–Pérot-type organic microcavity incorporating an organic semiconductor active layer, and demonstrate polariton condensation within this platform. The work addresses an important practical challenge in organic polaritonics, where high-refractive-index dielectric mirrors are typically fabricated using vacuum deposition or sputtering processes. By implementing a fully solution-processed approach, the authors aim to provide a potentially more cost-effective and scalable route toward organic microcavity devices. Furthermore, the revised manuscript significantly strengthens the evidence for polariton condensation. The inclusion of TMM modelling, additional experimental data, and more detailed analysis collectively provides a more comprehensive and convincing dataset.*

The demonstration of a robust, fully solution-processed platform is of interest and may provide useful guidance for future device design and fabrication strategies. However, given the broad scope of Nature Communications and the fact that fully spin-coating-processed microcavities have already been reported in previous studies (e.g., Adv. Optical Mater. 2017, 5, 1700523; Nanoscale, 2019, 11, 8978), it would be important to more clearly demonstrate the physical property part, for example, why novel physical insights like ring-shaped condensation are uniquely enabled by this fabrication approach.

I provide the following comments for the authors' consideration.

Authors' response

We thank the Reviewer for this thoughtful and constructive assessment of our work. We are particularly grateful for the Reviewer's recognition that the manuscript addresses an important practical

challenge in organic polaritonics, and that the revised version now provides a substantially stronger and more convincing case for polariton condensation through the inclusion of TMM modelling, additional experimental data, and more detailed analysis. We also appreciate the Reviewer's positive view that the fully solution-processed platform may provide useful guidance for future device design and fabrication strategies.

We agree that the distinction from earlier fully spin-coated microcavity studies should be stated clearly. We did not intend to imply that fully spin-coated microcavities had not been reported previously. Rather, the advance of the present work lies in demonstrating a fully solution-processed organic microcavity platform that reaches polariton lasing / condensation in the strong-coupling regime, together with access to a reversible high-density redistribution of the condensate. In this sense, the significance of the manuscript lies not in fabrication alone, but in the combination of an experimentally accessible platform with physically interesting nonlinear condensate behaviour.

We also agree that the wording should avoid implying that the ring-shaped redistribution is uniquely or exclusively caused by the fabrication route itself. Our intention is instead to emphasize that the present platform enables reliable access to a high-density strong-coupling regime in which this behaviour can be observed and studied. We have therefore revised the manuscript to make this distinction clearer, while the detailed physical discussion is addressed in the specific responses below.

Finally, we thank the Reviewer for drawing attention to the earlier reports of fully spin-coated microcavities. In the revised manuscript, we have ensured that these studies are cited appropriately (e.g., Adv. Optical Mater. (2017), 5, 1700523; Nanoscale (2019), 11, 8978) and that the distinction from the present work is made more explicit. We have added the references pointed out by the reviewer and modified the following sentence from "~~To~~ our knowledge, no conventional optically pumped organic vertical-cavity lasers have yet been realized using solution-based methods." to "Although a few fully solution-processed microcavities have been demonstrated, (Lova, P. & al. Advanced Optical Materials 2017, 5, 1700523; Lova, P. & al. Nanoscale 2019, 11, 8978–8983) to our knowledge, no conventional optically pumped organic vertical-cavity lasers have yet been realized using solution-based methods."

Comment 1

The manuscript shows a ring-shaped emission profile at high excitation densities. It is not clear why this feature has not been observed in previous studies, and why it appears in the present fully spin-coated microcavity.

One possibility is that higher excitation densities can be reached in this system, for example due to lower threshold or better stability compared to conventional cavities, leading to higher polariton densities.

It would be helpful if the authors could clarify this point. A comparison with microcavities fabricated by conventional methods (such as sputtering) may also help to understand the origin of this behaviour.

Answer

We thank the Reviewer for this insightful comment. We agree that this point deserves clarification. We do not wish to claim that the ring-shaped redistribution is uniquely caused by solution processing alone. Rather, our view is that the present fully solution-processed platform is particularly well suited to reveal this behaviour because it combines several favorable factors: a carefully optimized DBR design with high reflectivity obtained using a relatively small number of pairs, a well-matched active material/cavity combination, and reliable access to a high-density strong-coupling regime in which the condensate can be driven well above threshold while retaining the lower-polariton character.

We also agree with the Reviewer's suggestion that access to higher excitation density is likely to be part of the explanation. In the revised manuscript, we therefore clarify that the observed annular redistribution should not be interpreted as a generic consequence of spin coating itself, but rather as a behaviour that becomes experimentally accessible in the present platform because the cavity can be driven reproducibly into the relevant high-density regime.

A comparison with more conventional cavity fabrication routes is indeed informative. For example, the DPAVB-based microcavity study by Lidzey's group (McGhee et al., J. Mater. Chem. C 2022, 10, 4187–4195) reported polariton condensation in a cavity fabricated using more conventional mirror architectures, but did not report the annular redistribution behaviour observed here. More broadly, our own prior (Nat. Mater., 2014, 13, 271–278) and more recent work (Adv. Optical Mater. 2025, 13, 2500155) showed that sputtering can irreversibly damage some organic emitters to the extent that strong coupling is lost entirely, leaving only the cavity mode. This motivates our view that a gentler, fully solution-based fabrication route can be important for preserving the optical response of the active layer and thereby enabling access to high-density polaritonic behaviour. While this does not by itself prove the microscopic origin of the ring-shaped redistribution, it supports our view that preserving the active material and maintaining strong coupling at high density are important ingredients in making this behaviour observable.

To reflect this more carefully, we have also revised the manuscript text to avoid implying that the ring-shaped redistribution is an exclusive consequence of the fabrication route. Instead, we now frame it as a high-density condensate behaviour that becomes accessible in the present robust fully solution-processed platform.

In the introduction we changed the text from "~~Above threshold, the condensate also undergoes a spatial and momentum redistribution driven by repulsive polariton–polariton and polariton–reservoir interactions, reflecting the interacting nature of the condensate.~~" to "More importantly, the present platform enables polariton lasing with reliable access to a high-density regime, in which the condensate undergoes a reversible spatial and momentum redistribution. This behaviour is most clearly manifested in the emergence of a reversible annular condensate and is consistent with repulsive interaction-driven dynamics, with likely contributions from both polariton–polariton and polariton–reservoir interactions."

Comment 2

Considering that exciton diffusion typically occurs via intermolecular interactions, a neat film of the active material would be ideal. However, in this study, the dye is dispersed in a PS matrix.

It should be interesting to know whether increasing the dye concentration, or approaching a neat-film-like condition, would affect the observed ring-shaped redistribution, such as its spatial extent or dynamics.

Answer

We thank the Reviewer for this insightful suggestion. Indeed, increasing the dye concentration could increase the excitonic fraction relative to our original measurements and may therefore help further elucidate the underlying physics.

Following the Reviewer's suggestion, we fabricated new microcavity samples with twice the DPAVB concentration used in the polariton-lasing samples reported in the manuscript. Under otherwise identical excitation conditions, these higher-concentration samples did not exhibit polariton lasing. As shown in the response-letter figure Fig. R2, the emission intensity increased approximately linearly with pump power (Fig. R2a), while the linewidth broadened from about 4 nm to above 8 nm at higher excitation densities (Fig. R2b). In addition, the films showed visibly poorer optical quality, consistent with aggregation in the active layer.

We agree that this is a very interesting direction and one that we intend to pursue further in future work. At present, however, we are limited by the fact that lasing was not observed at

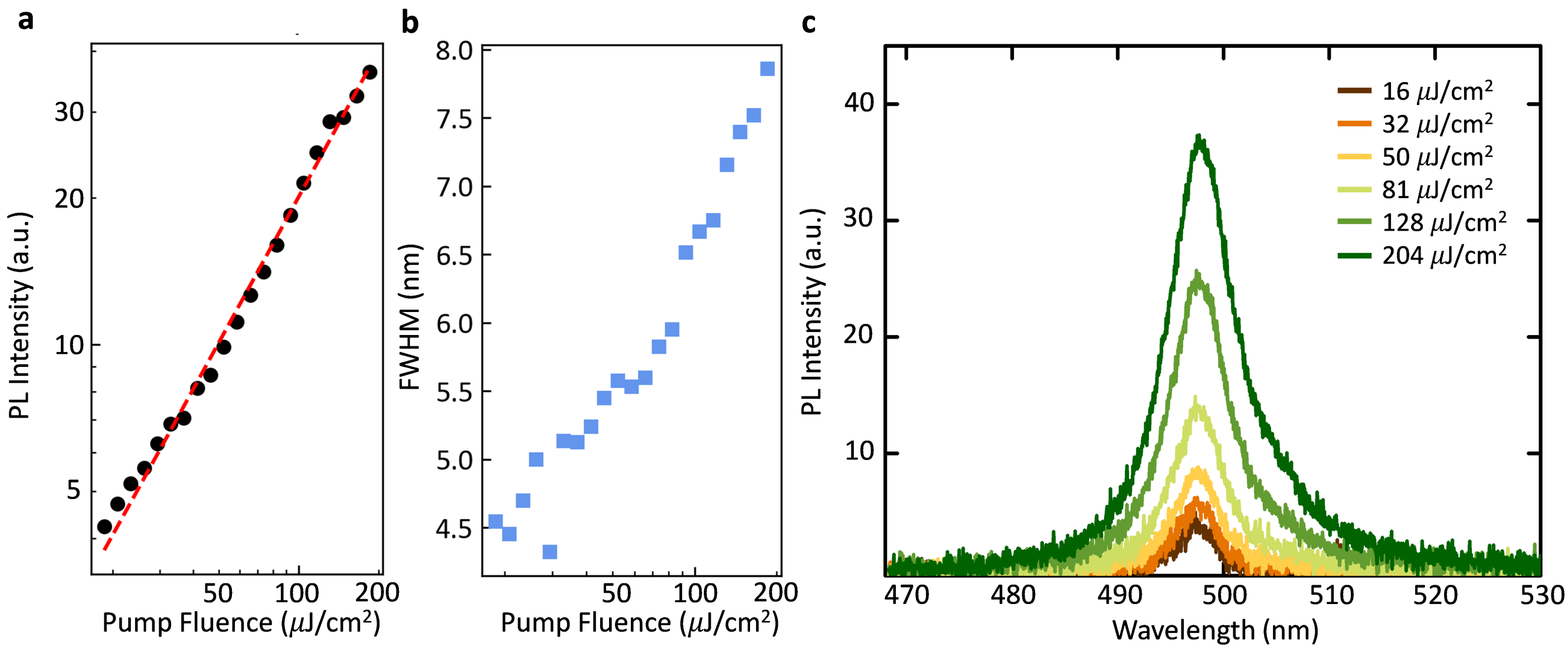

Fig. R2: Power-dependent photoluminescence of the high-concentration (40 mg/ml) DPAVB microcavity. (a) Integrated PL intensity as a function of pump fluence. The emission increases linearly with excitation density and is well described by a linear fit (red dashed line), indicating the absence of clear nonlinear threshold behavior. (b) Emission linewidth (FWHM) as a function of pump fluence, showing a continuous broadening with increasing excitation density rather than the linewidth narrowing expected for polariton lasing. (c) PL spectra recorded at increasing pump fluence, showing a gradual increase in emission intensity without abrupt spectral narrowing or mode collapse. The cavity is nominally similar to the device shown in Figure 3a, which exhibits polariton lasing at $\sim 20\ \mu J/cm^2$, indicating that the increased DPAVB concentration suppresses the lasing behavior and results in conventional spontaneous emission broadening.

the higher concentration, and therefore the annular emission could not be evaluated under those conditions. While it is difficult at this stage to determine the precise reason why lasing is suppressed, the degraded film quality suggests that aggregation and the resulting deterioration of the optical quality may play an important role.

To make this point explicit, we have added the following sentence to the Results section: "Moreover, polariton lasing was not observed in samples with similar Q but twice the DPAVB concentration."

Comment 3

What polariton density is needed to induce the ring-shaped diffusion? Would it be more appropriate to define the threshold in terms of the polariton population rather than the pump power?

Answer

We thank the Reviewer for this important question. We agree that the polariton population is a physically meaningful quantity for discussing the onset of the annular redistribution. At the same time, in the present work the directly controlled experimental parameter is the absorbed pump fluence, whereas converting this into an absolute polariton occupation requires additional assumptions regarding the collection efficiency, cavity outcoupling, and the number of occupied states. For this reason, and consistent with common practice in the field, we retain the threshold fluence P_{th} as the primary experimentally reported parameter in the manuscript. This distinction between experimentally reported threshold fluence and inferred condensate population is also in line with the approach used in our earlier work (Daskalakis et. al.,(2014) Nat. Mater., vol. 13, no. 3, pp. 271–278).

Nevertheless, we can provide an order-of-magnitude estimate of the detected emitted population per state, as well as of the detected emitted areal density. Following the same type of estimate used in Daskalakis et. al.,(2014) Nat. Mater., vol. 13, no. 3, pp. 271–278, we start from the mea-

sured emitted pulse energy and divide by the photon energy at the condensate wavelength. For the present samples, the emitted pulse energy measured at $1.47P_{\text{th}}$ is 2.5 pJ at 497 nm, corresponding to approximately 6.3×10^6 detected photons per pulse. Since the emitted photon flux at threshold is about 40 times smaller, this gives approximately 1.6×10^5 detected photons per pulse at threshold. We then estimate the number of states within the detection cone using the same phase-space approach as in Daskalakis et. al.,(2014) Nat. Mater., vol. 13, no. 3, pp. 271–278, with a 40 μm emission diameter, an 11 mm detector aperture placed 17 mm from the sample, and $\lambda = 497$ nm, giving approximately 1.6×10^3 states. This yields a pulse-integrated detected population of order 10^2 photons per state at threshold.

Using the same 40 μm emission diameter, the corresponding emission area is approximately 1.26×10^{-5} cm^2, which gives a detected emitted areal density at threshold of order 1.3×10^{10} cm^{-2}.

In addition, Supplementary Fig. S12 shows that the ring-shaped redistribution is clearly developed by around $5P_{\text{th}}$. Combining the power dependence in Fig. 3 with Supplementary Fig. S5, we estimate that the detected population per state in this regime is approximately two orders of magnitude higher than at threshold, i.e. of order 10^4 photons per state, corresponding to a detected emitted areal density of order 10^{12} cm^{-2}.

We stress, however, that these values should be regarded only as lower-bound experimental estimates of the detected pulse-integrated emitted population per state and areal density, rather than as the instantaneous polariton occupation inside the cavity. Because of the substantial uncertainty associated with the required correction factors, we prefer not to emphasize this estimate in the main manuscript and instead retain absorbed pump fluence as the more direct and robust quantity for defining threshold and comparing measurements.

Comment 4

In this study, the excitation power can be increased to more than ten times the threshold. Is there any transition from polariton lasing to photon lasing under such conditions? For example, is there any features of a two-threshold behavior?

Answer

Indeed, this was one of the key issues we aimed to address in the previous revision, which motivated the addition of Supplementary Figs. S10 and S11 to analyze the high-excitation-density regime in more detail. At the same time, we agree that we did not state explicitly enough that. We have therefore made this point more explicit in the revised text. Within the excitation range investigated here, we do not observe evidence for a transition from polariton lasing to photon lasing, nor two-threshold behaviour. This conclusion is supported by the fact that the emission remains confined within the lower-polariton dispersion across the full pump range and does not evolve toward the bare cavity mode, as would be expected upon degradation of strong coupling. The detuning-dependent behaviour is likewise consistent with renormalization within the strong-coupling regime rather than cavity-mode behaviour.

In addition, the power-dependent input–output curve in Fig. 3a shows a single pronounced nonlinear threshold at approximately 20 μJ/cm^2, accompanied by linewidth narrowing, with no indication of a second threshold within the measured range. This is further supported by the corresponding power-dependent measurements shown in Supplementary Figs. S3, S5, and S7. To make this conclusion even more explicit, we have now added a new panel, Supplementary Fig. S12e, which shows the integrated PL intensity over the full excitation range investigated, extending up to $20P_{\text{th}}$. This plot does not reveal any second nonlinear threshold. Rather, when the emission redistributes spatially and in momentum space at high pump powers, the integrated emission tends to saturate instead of showing the emergence of a second threshold.

Accordingly, we added the following description to the caption of Supplementary Fig. S12:“

(e) The integrated PL intensities over the full excitation range used in this and Supplementary Fig. S11.”.

We also added the following sentence to the caption of Supplementary Fig. S11: “The integrated PL intensities over the full excitation range are shown in Supplementary Fig. S12(e).”

We therefore interpret the observed high-density regime as remaining within strong coupling over the excitation range studied, rather than crossing into conventional photon lasing.

To make this point explicit in the main text, we changed the sentence “Across the full excitation range, the emission stays confined within the lower polariton dispersion and does not evolve toward the bare cavity mode” to “Across the full excitation range, the emission stays confined within the lower polariton dispersion and does not evolve toward the bare cavity mode, and no second threshold is observed that would indicate a transition to photon lasing (Supplementary Fig. S12e).”

Comment 5

The manuscript mentions scalability, but it is not clear what device size can realistically be achieved with the present approach.

Since spin coating typically has limitations in terms of large-area uniformity, it would be helpful if the authors could comment on the scalability of this method.

For example, could similar structures be fabricated using other solution-based techniques such as blade coating?

Answer

We agree that the term “scalability” should be used carefully in the present work. Our intention is not to claim that the current study demonstrates engineering-scale manufacturability, but rather that it establishes a fully solution-processed microcavity platform that is experimentally accessible and compatible with future scale-up strategies. Consistent with this point, we have softened the relevant forward-looking wording in the manuscript. In particular, in the Abstract, the sentence “Together, the fabrication approach and the observed lasing dynamics ~~establish a route toward scalable polaritonic and quantum photonic technologies~~ and provide new opportunities for studying nonlinear polariton physics in organic systems.” was revised to “Together, the fabrication approach and the observed lasing dynamics establish a viable platform for exploring future polaritonic and quantum photonic technologies and provide new opportunities for studying nonlinear polariton physics in organic systems.”

In the present study, our evidence is limited to the coated areas investigated experimentally, over which we observe smooth position-dependent detuning together with only modest variation in cavity quality factor and lasing threshold. We therefore view the present work as establishing feasibility on the laboratory scale studied here, rather than as a direct demonstration of large-area manufacturing.

We also agree that spin coating has inherent limitations with respect to large-area thickness uniformity. However, the broader significance of the present result is that both the DBRs and the active layer are processed entirely from solution. This means that, in principle, the same materials platform could be explored in the future using other solution-based coating methods that are more naturally suited to scale-up, such as blade coating or related meniscus-guided deposition approaches.

Comment 6

In typical microcavity structures for polariton formation, one side of the DBR is often designed to have a reflectivity close to 100%. In this study, however, the top DBR consists of 7.5 pairs with a reflectivity of about 94%.

It is not clear why this design was chosen. Would increasing the number of DBR pairs and achieving higher reflectivity improve the cavity performance or polariton properties?

Answer

We thank the Reviewer for this very useful technical comment. The choice of a 7.5-pair top DBR was deliberate and reflects a design optimization rather than a limitation of the approach.

First, for a quarter-wave DBR, the maximum reflectivity increases with the number of pairs according to

$$R_{\max} = \left(\frac{1 - \left(\frac{n_L}{n_H}\right)^{2N}}{1 + \left(\frac{n_L}{n_H}\right)^{2N}} \right)^2,$$

where n_H and n_L are the refractive indices of the high- and low-index layers and N is the number of DBR pairs. For the refractive-index contrast of our TiOH:PVA/Nafion DBRs, the reflectivity rises rapidly and approaches a plateau after approximately seven pairs. We therefore chose the smallest number of pairs that already yields very high reflectivity, in order to avoid unnecessary increases in total stack thickness. This is important because adding more pairs also increases cavity-field penetration into the DBRs, can promote stress build-up between the layers, can propagate interfacial roughness through the stack, and increases fabrication complexity and time. To make this point clearer, we have updated Supplementary Fig. S1 to include a new panel showing the theoretical reflectivity as a function of the number of DBR pairs. This new panel also shows that the experimentally obtained reflectivity is already very close to the calculated maximum for this material system. We have added into the main text the following reference to the new panel S1b: "Supplementary Fig. S1b shows how the calculated reflectivity plateaus after 7 pairs. The choice of 7.5 pairs therefore reflects a practical optimization, providing near-maximal reflectivity while limiting additional stack thickness, fabrication time, and other trade-offs associated with increasing the number of pairs further."

Second, we would like to distinguish between the conditions for strong coupling and the conditions for optimizing the polariton lasing threshold. For strong exciton–photon coupling, the commonly used condition is that the coupling strength exceeds the mean dissipative losses,

$$2g_N > \frac{\gamma_c + \gamma_m}{2},$$

rather than that one mirror must have reflectivity arbitrarily close to 100% (see review article DOI:10.1021/acs.chemrev.2c00895). In this sense, a partially transparent top mirror is entirely compatible with polariton formation and is often advantageous for practical optical access. Indeed, many polaritonic microcavities, including metal-clad structures, operate in the strong-coupling regime with substantially lower top-mirror reflectivity than a nearly ideal dielectric DBR.

If the Reviewer's question instead concerns polariton-lasing performance, then we agree that increasing the DBR reflectivity can in principle increase the cavity photon lifetime and thereby reduce the lasing threshold. This is also consistent with our previous work in Nature Materials (Daskalakis et. al.,(2014) Nat. Mater., vol. 13, no. 3, pp. 271–278), where we reported polariton lasing in organic microcavities with both 6-pair and 9-pair DBRs, and showed that increasing the number of DBR pairs increased the polariton lifetime, lowered the threshold fluence, and reduced the associated emission blueshift (Daskalakis et. al.,(2014) Nat. Mater., vol. 13, no. 3, pp. 271–278). It can also reduce top-side outcoupling, which is important for reflectivity and photoluminescence measurements. In the present system, a 7.5-pair top DBR already provides sufficiently high reflectivity ($\sim 94\%$) and a cavity quality factor $Q > 250$, enabling both clear strong coupling and polariton lasing while preserving sufficient top-side outcoupling for reflectivity and photoluminescence measurements. For potential applications of this platform, the top mirror could be made even more reflective (and hence potentially further lower the threshold), if top-side spectroscopic access is not required.

We hope the updated Supplementary Figure S1 and our clarification makes clear that the use of 7.5 pairs was a deliberate and well-motivated design choice.

Comment 7
The advantage of using polymer-based materials is their flexibility; is this microcavity device actually flexible?

Answer
We thank the Reviewer for this interesting question. The present microcavity devices are not flexible, as they are fabricated on quartz substrates. In principle, the polymer-based and solution-processed nature of the platform could make adaptation to flexible substrates possible in future work. However, for the present optically pumped polariton lasing experiments, flexible substrates are not ideal because the excitation wavelength used here (380 nm) is strongly absorbed by many common flexible substrate materials, which can lead to substrate degradation under the high pump fluences required. For this reason, quartz was used in the present study to ensure optical transparency and stability under excitation.

Yours sincerely,
The Authors

Response to the Reviewer report

A fully solution-processed organic microcavity laser in the strong light–matter coupling regime

Hassan A. Qureshi, Henri Lyyra, Akseli Korkeamäki, Oskar Tuomi, Antti J. Moilanen, and Konstantinos S. Daskalakis
Corresponding author: konstantinos.daskalakis@utu.fi

We thank the Editor for the opportunity to submit a further revised version of our manuscript and the Reviewer for the comments provided in this round of review. We have carefully considered the remaining points, which prompted us to clarify the technical aspects of the reflectivity measurements and to articulate more explicitly the experimental tunability and broader significance of the fully solution-processed planar DBR platform. Below, we provide a point-by-point response to all comments raised in this round. In the revised manuscript, all newly added text is shown in blue.

Reviewer #3 (Remarks to the Authors):

The author emphasizes that the significance of the manuscript lies not in either element considered separately, but in their combination. However, in my opinion, the fully solution-processed organic microcavity platform has been reported before (Adv. Mater. 2024, 36, 2212056). The reversible redistribution of the polariton condensate at high pump densities also has been reported (Nat. Commun. 2021, 12, 3265). The author only reorganized these existing works without substantial innovation, which does not meet Nature Communications' requirements for significant progress.

The reflectance spectra in microcavities are key evidence for the occurrence of strong exciton-photon coupling, and a prerequisite for the so-called exciton polaritons condensation and redistribution in this work. However, the author provides reflectance spectra at 55° and 60° in Figure R1, where UP, MP2 and LP can be seen, while MP1 is completely absent. I am confused why the MPB can only be observed at a large angle, and why there is no signal at a small angle?

This manuscript belongs to the incremental work in the field of organic polaritons, with no significant breakthroughs in both process and physics, and key scientific issues are not yet clear, which does not meet the publishing standards of Nature Communications. Suggest transferring to professional optical/optical journals.

Authors' response

We thank the Reviewer for this follow-up comment, which prompted us to revisit the previously cited Nature Communications paper (2021, 12, 3265) and to clarify more explicitly how the organic single-crystal microribbon cavities reported there differ from the fully solution-processed planar DBR platform studied here. In that system, the observed high-density redistribution is closely linked to propagation along the microribbon axis, where the dimensional confinement and waveguiding geometry naturally favor finite-wave-vector emission away from the excitation region.

As a platform, microribbon cavities are also distinct from our system because their optical geometry is largely determined by the spontaneous growth, dimensions, and morphology of the organic crystals, offering less direct control over the systematic variation of cavity parameters. A key advantage of our platform is precisely its ability to support controlled and systematic studies of polariton condensation. In the present manuscript, we exploit this flexibility by varying the cavity detuning

and, to a more limited extent, by examining a different DPAVB concentration in the additional measurement presented in our response to Reviewer 4. We therefore thank the Reviewer for making us realize that this important advantage of our platform was not sufficiently emphasized in the manuscript. We have now clarified this point in the manuscript by adding the following sentence to the Abstract: "By studying cavities with different detunings, we systematically investigate how the excitonic fraction of the polariton modes affects the outward redistribution of emission at high excitation densities.". We also revised the final sentence of the Abstract to emphasize the experimental tunability of the platform: "Together, the fabrication approach, experimental tunability, and observed lasing dynamics establish a viable platform for exploring future polaritonic and quantum photonic technologies and provide new opportunities for studying nonlinear polariton physics in organic systems." Finally, we added the following sentence to the Summary: "Comparison across cavities with different detunings further enables us to assess the role of polariton excitonic content in this high-density outward redistribution."

We also wish to clarify that the revised manuscript already cites the Advanced Materials (2024, 36, 2212056) work from the Stingelin group, as well as earlier fully spin-coated microcavity studies by Lova and Comoretto (Advanced Optical Materials 2017, 5, 1700523; Nanoscale 2019, 11, 8978–8983). We fully agree that these papers are important precedents for fully solution-processed organic microcavities. However, these works did not demonstrate polariton lasing/condensation from a fully solution-processed planar DBR microcavity. This distinction is important because demonstrating strong coupling in reflectance or transmission does not automatically imply that the same structure can exhibit measurable photoluminescence from a polariton state, let alone nonlinear polariton lasing. Indeed, the Advanced Materials 2024 study established an important fully solution-processed strongly coupled platform, characterized through reflectance, transmission, group delay, and transfer-matrix modelling, but did not report photoluminescence or nonlinear emission from the coupled modes. This underlines that reaching an emissive, high-density polariton regime in such a fully solution-processed planar architecture is a separate and technically non-trivial advance.

The significance of the work is therefore twofold: fundamentally, it provides access to interaction-driven condensate dynamics in a controllable planar geometry; as an enabling advance, it establishes an experimentally accessible route to polariton lasing that does not require advanced nanofabrication or vacuum-deposition infrastructure. We believe that this combination of fundamental insight and enabling platform development constitutes the substantive advance of the present work.

We thank the Reviewer for giving us the opportunity to further clarify this technical aspect of the reflectivity measurements and the visibility of the middle polariton branches (MPBs). To directly address why the MPBs are only resolved at larger angles, and why they are not clearly visible at smaller angles, we have included an additional Response Figure R1. In reflectance, the visibility of a given polariton branch is governed not only by its presence in the coupled eigenmode structure, but also by the optical contrast with which it appears in the measured spectrum. In the present measurements, the reflectivity spectra were recorded using s-polarized light. As shown in Response Figure R1, the overall reflectivity of the microcavity for s-polarization decreases substantially with increasing incidence angle. Specifically, the maximum reflectivity is close to 90% at 15°, whereas it decreases to below 80% at 55°. As a result, weak middle-branch resonances that are masked by the high-reflectivity background at small angles become easier to resolve at larger angles.

This explains why the middle-polariton feature that is experimentally discernible in Figure R1 is observed at larger angles rather than at small angles. We stress that the absence of a clearly resolved reflectance dip at small angles should not be interpreted as the absence of the corresponding coupled mode. Rather, under the present s-polarized reflectance conditions, the optical contrast of the middle branches is limited, and only features with sufficient contrast relative to the local reflectivity background can be resolved directly in the experimental spectra. Importantly, the polariton

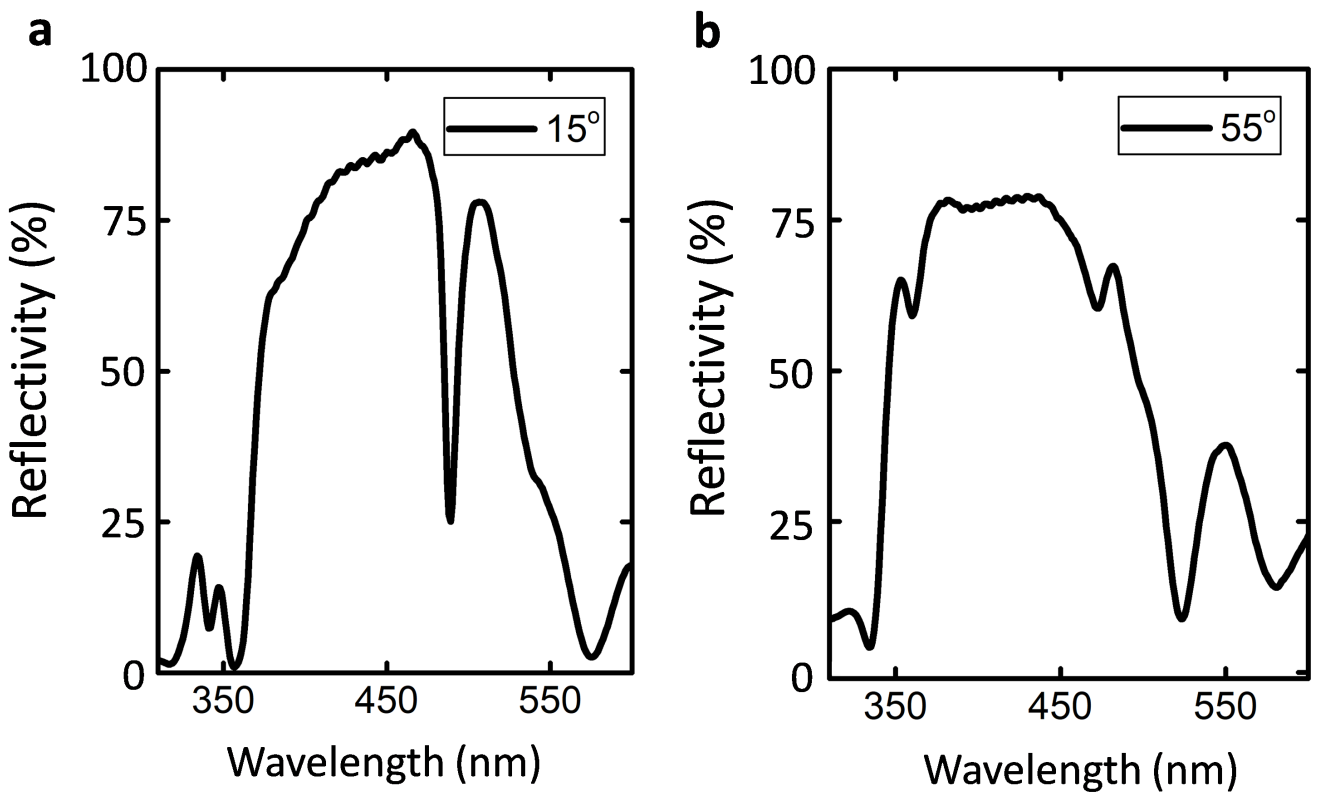

Fig. R1: Angle-dependent reflectivity spectra of the solution-processed polariton microcavity recorded at 15° and 55°. a) At 15°, the cavity shows a high reflectivity background of approximately 90%, with a pronounced reflectivity minimum near the polariton/cavity resonance. b) At 55°, the overall reflectivity background decreases to approximately 79%, showing weakly resolved MP.

branch assignment is not based solely on the visual visibility of individual reflectance minima. The coupled eigenmode structure is supported by a physically constrained analysis using the measured refractive indices of the DPAVB:PS films, together with excitonic oscillator parameters extracted from the refractive-index analysis of the DPAVB:PS layer. The coupled-harmonic-oscillator model then provides the expected polariton branches, while the measured reflectance spectra determine which of these branches have sufficient contrast to be observed experimentally under the present s-polarized conditions.

Following the Reviewer's comment, we also realized that the polarization condition of the reflectivity measurements had not been stated sufficiently clearly. We have therefore added the following sentence to the Methods section: "All angle-resolved reflectivity measurements reported here were performed using s-polarized incident light."

Yours sincerely,
The Authors

Qureshi et al. report an organic exciton polariton laser microcavities fabricated entirely by solution processing, which includes both organic molecules and DBR prepare by solution method. And reversible, interaction driven redistribution of polariton lasing is observed at a high excitation density, revealing significant polariton laser behavior in organic microcavities. However, I don't believe that the all-solution device manufacturing method and the flowable exciton polariton laser are highly innovative. Therefore, I don't recommend this work publishing in Nature Communications. It is more suitable for other professional journals.

1. The author claims that one of the innovations of this manuscript is the preparation of organic films and DBR through a full-solution method, but no relevant data is provided on the uniformity of DBR, which makes it difficult for readers to believe that large-area, high reflectivity DBR can be obtained through a simple spin-coating method. The author should provide a cross-sectional view of the thickness of DBR within the microscopic region, such as scanning electron microscope (SEM) imaging, to confirm the thickness and uniformity of each layer of DBR medium throughout the entire size range.

2. The angle-dependent reflectivity in Figure 2b is the most important data to demonstrate strong exciton-photon coupling. The author claims to have seen the LPB, MPB and UPB. However, the MPB are not seen in the Figure 2b, and the author needs to provide more intuitive and clear evidence. In addition, how are the three exciton peak positions of DPAVB determined? The author needs to provide the corresponding absorption spectra of pure DPAVB films with the same thickness as in Figure 2b.

3. Figure 4 shows that after increasing the pump density, the condensate redistributes outward as it gains momentum, forming an annular emission profile due to the repulsion of polariton-reservoir and polariton-polariton interactions. However, according to previous reports, the blue-shift mechanism of polariton energy in organic semiconductor microcavities is different from that in inorganic semiconductor microcavities. The energy blue-shift in organic semiconductor microcavities is due to the interplay of the saturation effects and intermolecular energy migration, or the phase space filling effects, rather than the interaction between polaritons (*Comm. Phys.* 2020, 3(1), 18; *ACS Photon.* 2019, 7(2), 384-392). The author should reconsider the physical mechanism of redistribution of polariton towards higher energy.

4. On page 11 of the manuscript, it is described as "As shown in Supplementary Fig. S6, neat DPAVB:PS films exhibit irreversible damage under ASE cycling, whereas the polariton microcavities can be repeatedly driven across threshold without any change in emission profile or intensity (Supplementary Fig. S7)". I don't understand why the optical stability of DPAVB films in microcavities is enhanced. As far as I know, organic semiconductors are generally unstable, especially after high-power pump density irradiation, their stability decreases and is accompanied by an increase in the condensation threshold. However, the condensation data in Figure S7 is surprisingly stable. Can the author explain why the optical properties of the film are so stable?

Overall, although the work in this manuscript is interesting, its innovation is

insufficient, and the physical mechanism of the redistribution of polariton energy remains to be discussed. So I don’t recommend publishing it in Nature Communications.

The author emphasizes that the significance of the manuscript lies not in either element considered separately, but in their combination. However, in my opinion, the fully solution-processed organic microcavity platform has been reported before (Adv. Mater. 2024, 36, 2212056). The reversible redistribution of the polariton condensate at high pump densities also has been reported (Nat. Commun. 2021, 12, 3265). The author only reorganized these existing works without substantial innovation, which does not meet Nature Communications' requirements for significant progress.

The reflectance spectra in microcavities are key evidence for the occurrence of strong exciton-photon coupling, and a prerequisite for the so-called exciton polaritons condensation and redistribution in this work. However, the author provides reflectance spectra at 55° and 60° in Figure R1, where UP, MP2 and LP can be seen, while MP1 is completely absent. I am confused why the MPB can only be observed at a large angle, and why there is no signal at a small angle?

This manuscript belongs to the incremental work in the field of organic polaritons, with no significant breakthroughs in both process and physics, and key scientific issues are not yet clear, which does not meet the publishing standards of Nature Communications. Suggest transferring to professional optical/optical journals.
